# World Health Organization Estimates of the Relative Contributions of Food to the Burden of Disease Due to Selected Foodborne Hazards: A Structured Expert Elicitation

**DOI:** 10.1371/journal.pone.0145839

**Published:** 2016-01-19

**Authors:** Tine Hald, Willy Aspinall, Brecht Devleesschauwer, Roger Cooke, Tim Corrigan, Arie H. Havelaar, Herman J. Gibb, Paul R. Torgerson, Martyn D. Kirk, Fred J. Angulo, Robin J. Lake, Niko Speybroeck, Sandra Hoffmann

**Affiliations:** 1 Technical University of Denmark, Lyngby, Denmark; 2 Aspinall & Associates, Tisbury, England; 3 Bristol University, Bristol, England; 4 Ghent University, Merelbeke, Belgium; 5 Université catholique de Louvain, Brussels, Belgium; 6 Institute of Tropical Medicine, Antwerp, Belgium; 7 Resources for the Future, Washington, District of Columbia, United States of America; 8 Technical University of Delft, Delft, the Netherlands; 9 World Health Organization, Geneva, Switzerland; 10 National Institute for Public Health and the Environment, Bilthoven, the Netherlands; 11 University of Florida, Gainesville, Florida, United States of America; 12 Utrecht University, Utrecht, Netherlands; 13 Gibb Epidemiology Consulting LLC, Arlington, Virginia, United States of America; 14 University of Zurich, Zurich, Switzerland; 15 The Australian National University, Canberra, Australia; 16 U.S. Centers for Disease Control and Prevention, Atlanta, Georgia, United States of America; 17 Institute of Environmental Science and Research, Christchurch, New Zealand; 18 U.S. Dept. of Agriculture, Economic Research Service, Washington, District of Columbia, United States of America; University of Minnesota, UNITED STATES

## Abstract

**Background:**

The Foodborne Disease Burden Epidemiology Reference Group (FERG) was established in 2007 by the World Health Organization (WHO) to estimate the global burden of foodborne diseases (FBDs). This estimation is complicated because most of the hazards causing FBD are not transmitted solely by food; most have several potential exposure routes consisting of transmission from animals, by humans, and via environmental routes including water. This paper describes an expert elicitation study conducted by the FERG Source Attribution Task Force to estimate the relative contribution of food to the global burden of diseases commonly transmitted through the consumption of food.

**Methods and Findings:**

We applied structured expert judgment using Cooke’s Classical Model to obtain estimates for 14 subregions for the relative contributions of different transmission pathways for eleven diarrheal diseases, seven other infectious diseases and one chemical (lead). Experts were identified through international networks followed by social network sampling. Final selection of experts was based on their experience including international working experience. Enrolled experts were scored on their ability to judge uncertainty accurately and informatively using a series of subject-matter specific ‘seed’ questions whose answers are unknown to the experts at the time they are interviewed. Trained facilitators elicited the 5^th^, and 50^th^ and 95^th^ percentile responses to seed questions through telephone interviews. Cooke’s Classical Model uses responses to the seed questions to weigh and aggregate expert responses. After this interview, the experts were asked to provide 5^th^, 50^th^, and 95^th^ percentile estimates for the ‘target’ questions regarding disease transmission routes. A total of 72 experts were enrolled in the study. Ten panels were global, meaning that the experts should provide estimates for all 14 subregions, whereas the nine panels were subregional, with experts providing estimates for one or more subregions, depending on their experience in the region. The size of the 19 hazard-specific panels ranged from 6 to 15 persons with several experts serving on more than one panel. Pathogens with animal reservoirs (e.g. non-typhoidal *Salmonella* spp. and *Toxoplasma gondii*) were in general assessed by the experts to have a higher proportion of illnesses attributable to food than pathogens with mainly a human reservoir, where human-to-human transmission (e.g. *Shigella* spp. and Norovirus) or waterborne transmission (e.g. *Salmonella* Typhi and *Vibrio cholerae*) were judged to dominate. For many pathogens, the foodborne route was assessed relatively more important in developed subregions than in developing subregions. The main exposure routes for lead varied across subregions, with the foodborne route being assessed most important only in two subregions of the European region.

**Conclusions:**

For the first time, we present worldwide estimates of the proportion of specific diseases attributable to food and other major transmission routes. These findings are essential for global burden of FBD estimates. While gaps exist, we believe the estimates presented here are the best current source of guidance to support decision makers when allocating resources for control and intervention, and for future research initiatives.

## Introduction

Foodborne diseases (FBD) are an important cause of morbidity and mortality worldwide [1, 2, 3]. The human health burden due to contaminated food is currently unknown. In order to fill this knowledge gap, the Foodborne Disease Burden Epidemiology Reference Group (FERG) was established in 2007 by the World Health Organization (WHO) to estimate the global burden of FBD.

Estimating the burden of FBD is complicated because most of the hazards causing foodborne disease are not transmitted solely by food. The relative impact of each route differs depending on the epidemiology of the disease causing microorganism (bacteria, virus or parasite) or chemical hazards. Other factors such as the geographical region, season, and food consumption patterns also influence the role of different exposures routes [4, 5]. The estimation of the burden of FBD, therefore, requires a delineation of the major transmission routes including contaminated food, water, soil, air, or contact with infected animals or humans. Previous efforts to quantify the contribution of specific sources (including types of foods) and transmission routes have been gathered under the term ‘source attribution’ or ‘human illness attribution’ [6, 7]. The applicability of available methods for source attribution of FBD at the global level was recently assessed by Pires [4].

Knowledge of the most important sources of exposure can foster better, more targeted control measures, and support risk managers in decisions on allocating resources to achieve the greatest public-health benefits. Source attribution is an important tool for identifying and prioritizing effective interventions to prevent and control FBD [8]. The need for reliable source attribution estimates has prompted a growing body of research focusing on attribution, particularly for infectious agents [4, 7, 9, 10]. However, comprehensive attribution studies based on surveillance data and/or food monitoring and exposure data are still limited in scope, and to date have been performed for a few hazards only or in a limited number of countries [11, 12, 13, 14, 15, 16, 17, 18, 19, 20, 21, 22, 23]. In addition, existing studies have focused mainly on identifying specific food sources or animal reservoirs, whereas other potential transmission routes are often not quantified due to lack of data or neglected due to the complexity of attribution models. Many studies, often designed as randomized controlled intervention trials, have been conducted to assess the importance of water, particularly for the transmission of diarrheal diseases (reviewed by [24, 25]). However, other transmission routes, such as soil, air and direct contact with infected humans or animals, are generally not considered in those studies. Thus, for most countries, and at the global level, relevant studies and data for quantifying attribution of potential FBD to the major transmission routes do not exist.

In such situations, structured elicitation of scientific judgment may be used [4, 26]. When data are not available, or undertaking primary research is not feasible, a structured elicitation offers a transparent and mathematically rigorous way of evaluating and enumerating uncertainty distributions from the judgments of many individual researchers, for quantifying risk models. Scientific judgment elicitations have been used in several risk domains including climate change [27, 28, 29, 30], critical infrastructure [31], genetically modified organisms [32], and volcanic risks [33, 34, 35, 36]. Within food safety, the approach has been applied to provide national estimates for the proportion of illnesses attributable to food for specific infectious diseases [37, 38, 39, 40, 41, 42, 43, 44], or to inform modeling of foodborne disease risk assessment models by estimating specific model parameters and their uncertainty [45, 46].

In this paper, we describe an expert elicitation study conducted by the FERG Source Attribution Task Force (SATF) to estimate the relative contribution of food and other major transmission pathways to the global burden of diseases commonly transmitted by food. The resulting estimates were used as input to the disease burden framework developed by the FERG Computational Task Force [47] to estimate the global burden of FBD [48].

## Materials and Methods

**Fig 1:**
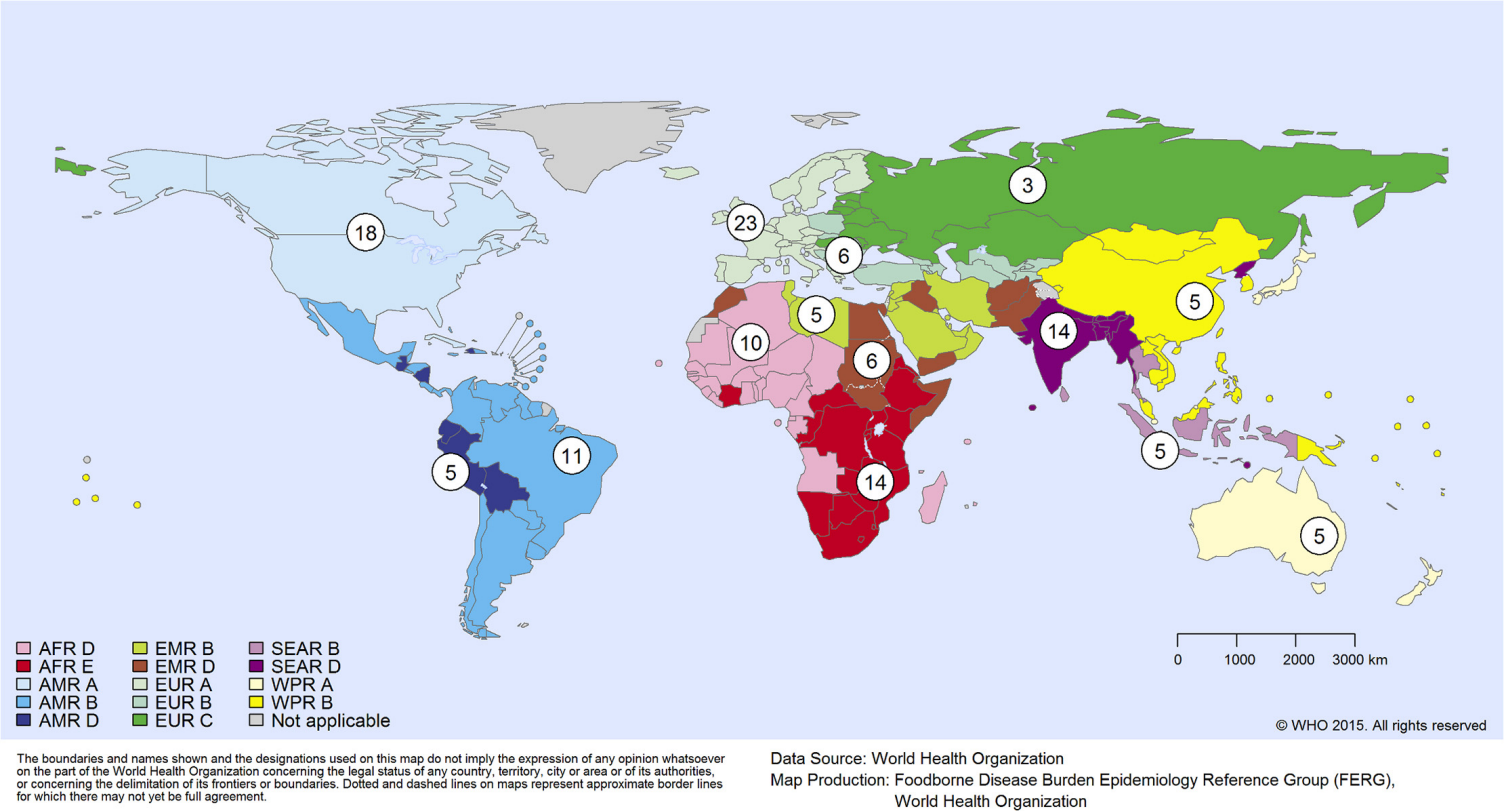
Geographical distribution of the number of experts per subregion according to working experience (>3 years). **Several experts had experience in more than one subregion.** The subregions are defined on the basis of child and adult mortality as described by Ezzati et al. [63]. Stratum A: very low child and adult mortality, Stratum B: low child mortality and very low adult mortality, Stratum C: low child mortality and high adult mortality, Stratum D: high child and adult mortality, and Stratum E: high child mortality and very high adult mortality. AFR = African Region; AMR = Region of the Americas; EMR = Eastern Mediterranean Region; EUR = European Region; SEAR = South-East Asia Region; WPR = Western Pacific Region. A list of countries per subregions can be found in Havelaar et al. (48). The use of the term ‘subregion’ here and throughout the text does not identify an official grouping of WHO Member States, and the ‘subregions’ are not related to the six official WHO regions.

Overall, the study was designed to provide estimates of the percent of illness acquired through different major routes of exposure. Major exposure routes considered were: food, environmental (water, soil, air), human-to-human transmission, direct animal contact and a variety of potential lead exposure sources. Exposure route attribution estimates were developed for 19 individual hazards for each of the fourteen subregions (Table 1, Fig 1). Three hazard-based task forces within the FERG, the Enteric Diseases Task Force, the Parasitic Diseases Task Force, and the Chemicals and Toxins Task Force identified, from their prioritized lists of hazards, those to be included in the expert elicitation [49, 50, 51]. Certain hazards were considered 100% foodborne, i.e., *Listeria monocytogenes*, *Mycobacterium bovis*, all foodborne trematodes, *Taenia solium*, *Trichinella* spp., cyanide in cassava and peanut allergens. For aflatoxin, inorganic arsenic, cadmium, dioxin, and methyl mercury, the Chemicals and Toxins Task Force determined that adequate data on foodborne exposure existed to use a risk assessment approach for estimating the foodborne disease burden, thus negating the need for attribution. The remaining hazards were included in the structured expert elicitation (Table 1).

**Table 1:** Foodborne hazards and structure of the expert panels.

Hazard groups	Hazards	Panel structure^a^	No. of panels
**Diarrheal disease**			
** **Bacteria	*Campylobacter* spp., enteropathogenic *Escherischia coli* (EPEC), enterotoxigenic *E*. *coli* (ETEC), Shiga toxin-producing *E*. *coli* (STEC), non-typhoidal *Salmonella* spp., *Shigella* spp., *Vibrio cholerae*	Subregional	7
** **Virus	Norovirus	Subregional	1
** **Intestinal protozoa	*Cryptosporidium* spp., *Entamoeba histolytica*, *Giardia* spp.	Global	3
**Other infectious disease**			
** **Bacteria	*Brucella* spp., *Salmonella* Typhi	Global Subregional	2
** **Virus	Hepatitis A virus	Global	1
** **Protozoa	*Toxoplasma gondii*	Global	1
** **Helminths	*Ascaris* spp., *Echinococcus granulosus*, *Echinococcus multilocularis*	Global	3
**Chemicals**			
** **Lead		Global	1
**Total**			19

^a^ Experts on a global panel were asked to provide estimates for all 14 subregions, whereas experts on a sub-regional panel could choose a set of subregions depending on their expertise.

### Identification of experts

An iterative peer nomination process based on a professional network sampling technique, sometimes called ‘snowball sampling’ was used to identify a pool of potential expert participants for this study. The first points of contact were identified through FERG members and other networks (e.g. Global Foodborne Infections Network—GFN, Global Environment Monitoring System—GEMS, International Network of Food Safety Authorities—INFOSAN, Joint FAO/WHO Expert meeting on Microbial Risk Assessment—JEMRA, Joint FAO/WHO Expert Committee on Food Additives—JECFA, European Food Safety Authority’s (EFSA) scientific panels, and WHO regional food safety advisors). These persons were asked to use their professional contacts and recognized expertise in relevant areas to nominate additional experts. Since the purpose of this process was to identify an adequately large pool of appropriate experts, rather than to identify the entire expert network, the process of referral continued until an adequate size pool was identified to fill panels of typically 8 to 12 experts per panel.

### Selection of experts

In collaboration with the hazard-based task forces of the FERG, the SATF defined a set of criteria for inclusion of experts. These criteria considered the experts’ background (education, and current and past positions), years of experience within the field and geographic coverage of expertise within the panel. The WHO invited nominated experts to participate, and the experts were asked to complete a declaration of interests (DOI) and an expert sheet providing information on their research/working area, highest education, current position, geographical experience, and years of experience. The experts were asked also to indicate which panel(s) they believed themselves suited for. The experts were not offered any compensation for their participation. The chairs of the three hazard-based task forces and the SATF reviewed the experts’ information and CVs and a final selection was made. FERG task force chairs and members of the SATF were not eligible for the study. DOI’s were evaluated by WHO.

Given the broad nature of the attribution task, care was taken to include a suitably wide range of scientific backgrounds and professional experience, and to ensure adequate geographical representation. Frequently, expert elicitations use publication record as the measure of recognized expertise [52]. However, in this study, restricting expert selection to choices based solely on publication records would have eliminated important groups of experts, in particular public-health field workers and food-safety professionals in developing countries.

### Expert panels

The panels for *Brucella* spp., Hepatitis A virus, parasitic diseases including intestinal protozoa and lead were structured as global panels, meaning that all experts in those panels were asked to provide estimates for all fourteen subregions (Table 1, Fig 1). The panels for the eight bacterial and viral pathogens causing diarrhea and *Salmonella* Typhi were structured as sub-regional panels, *i*.*e*., separate panels were created for each subregion. In general, experts on all panels were free to decide the subregions and/or hazards for which they provided their judgments.

### Analytical method

The study used Cooke’s Classical Model for expert elicitation [52, 53, 54]. This approach uses calibration or seed questions to develop performance weights used in aggregating experts’ judgments. The paradigmatic seed question is one for which the true value is not known at the time the experts answer the question, but will be known or is expected to become known post hoc. Thus the experts are not expected to know these values, but should be able to capture a majority of them reliably by defining suitable credible intervals.

Analysis of the experts’ performance on the seed variables has two main purposes: 1) to evaluate the expert’s *statistical accuracy* when assigning values to probability outcomes against the seed values (i.e., how reliably the expert’s credible interval responses capture the true values of the seed variables, statistically), and 2) to evaluate the expert’s *informativeness* when providing uncertainty distributions over the seed variables (i.e. how concentrated (narrow) are the distributions provided). Experts are thus scored with regard jointly to statistical accuracy (calibration score) and informativeness (information score). The statistical accuracy is measured as the p-value at which one would falsely reject the hypothesis that the expert’s probability assessments were statistically accurate), and informativeness is measured as Shannon relative information with respect to a user supplied background measure. Informativeness scores are not absolute, but relative to a set of experts assessing the same variables. The calculated calibration and information scores are used to aggregate experts’ judgments on target variables. The same measures can be applied to any combination of the experts' assessments to implement criteria for aggregating the assessments.

Cooke’s Classical Model provides a rigorous, quantitative means for estimating model parameters and their uncertainties and is the only elicitation procedure that has objective empirical control on expert scoring. Moreover, it allows formal optimization of aggregated uncertainty distributions in terms of statistical accuracy and informativeness [53]. The expert judgment processing software EXCALIBUR (http://www.lighttwist.net/wp/excalibur) also allows direct comparison of the results that would be obtained from unweighted aggregation of expert judgments with those produced by weighted linear pooling (or other combination schemes). For a more detailed description of the Classical Model see (S1 File) that also provide a list of references for different applications.

### Seed questions

It is not always possible to develop seed questions that are in the paradigmatic form of asking about a future event or measurement that has not been made, but could be made, in principle. The essential feature of a viable seed question is that the expert is not expected to know the exact value but, if they are a subject-matter expert, should be able to define a narrow uncertainty range that captures the value. Therefore, an alternative is to ask about selected data or values in the topic domain, about which the expert will not have perfect knowledge, nor access to realization values at the time they are answering the seed questions–but, for which the values are known to the analyst. Such retrospective questions are frequently used in expert elicitations applying the Cooke Classical Model (see e.g. [30, 38]).

In the present case, the seed questions we formulated were a mixture of retrospective and prospective seed variables. It is possible that expert uncertainty judgments vary by subject matter domain. In this study, the possibility of such biases relevant to foodborne illness source attribution was of concern. Therefore, the seed questions were designed to focus on questions that are substantively related to foodborne illness source attribution. Further, to account for the wide range of scientific backgrounds and experiences, seed questions covered a range of substantive topics relevant to source attribution. Five main categories of seed questions were identified for the panels on biological hazards (diarrheal pathogens and parasites): 1) dietary patterns and food supply; 2) under 5 years mortality rate; 3) access to improved water and sanitation; 4) disease surveillance, and 5) systematic reviews related to these and other scientific topics relevant to source attribution. For the panel on lead, questions were categorized as: 1) mean blood levels; 2) dietary exposure, and 3) dietary patterns and food supply. Examples of seed questions are presented in Table 2. All seed questions are provided in (S2 File).

**Table 2:** Examples of calibration seed questions.

Topic	Hazard	Question
Dietary patterns and food supply	All microbial hazards	Among all subregions in 2010 what was the proportion of regional vegetable supply (tonnes) that was imported rather than produced domestically in the subregion with the highest such percentage?
Under 5 mortality rate	*Brucella* spp., *Echinococcus* spp., intestinal protozoa, diarrheal pathogens	Based on WHO’s estimates think of the country in the African Region that had the largest percentage point decrease from 2000 to 2010 in all-cause under-5 mortality that was due to diarrhea. What was that percentage point decrease?
Disease surveillance	*Ascaris* spp., *Echinococcus* spp., intestinal protozoa, hepatitis A virus, diarrheal pathogens (developed subregions only)	What will be the rate per 100,000 population of laboratory confirmed human cases of campylobacteriosis in 2012 in all EU member states as reported in EFSA’s annual report?
Systematic review	All microbial hazards	Fewtrell et al (2005) conducted a systematic review and meta-analysis to compare the evidence of relative effectiveness of improvements in drinking water, sanitation facilities, and hygiene practices in less developed countries in reducing diarrheal illness. The meta-analysis of 5 studies was used to estimate the relative risk of diarrheal illness with and without multiple interventions. What was the estimated relative risk?
Mean blood level	Lead	What was the geometric mean blood lead concentration for all participants ages 1 year and older in the 2007–2008 U.S. NHANES survey? Please express your answer as positive micrograms per deciliter (μg/dL).

There were eight sets of seed questions with some overlapping questions for the biological hazards (S2 File), and a separate set for lead. This allows some consistency checks to be performed between panels on performance and scoring outcomes. The number of questions varied from ten to twelve per set of seed questions. Experts were asked to provide a central judgment in terms of the median value, and a 90% credible interval for each question. Seed questions were administered by facilitators through one-to-one telephone interviews. The experts were not presented with the seed questions before the interview and they were asked to provide estimates based on their experience, knowledge and judgment, without referring to other sources of information.

### Target questions

Target questions are the substantive questions of interest. In this study, for all identified hazards, we enquired about the percentage of all human disease cases caused by exposure through each of a number of indicated exposure routes. The point of exposure was chosen as the point-of attribution i.e. the experts were asked to distribute the disease burden on the sources that were the direct cause of human exposure. So for example, someone with a Norovirus infection might be exposed by eating food contaminated with the virus, although the food may have been contaminated by waste water at an earlier stage.

In order to reduce the time and effort burden of the elicitation on expert panelists, the hazard-based Task Forces decided which hazard exposure routes were relevant for present purposes (Table 3). For example, human-to-human transmission was excluded as an exposure route for *Brucella* spp. However, the questionnaires did provide experts with an option to indicate additional routes of transmission in case they disagreed with the Task Force’s evaluation.

**Table 3:** Exposure routes included in the expert elicitation. Hazards were grouped according to common exposure routes. na: not applicable, meaning that these exposure routes were considered not possible or extremely unlikely by the respective FERG task forces.

Hazard	Food	Animal Contact (Domestic and Wild)	Human to Human Contact	Water	Soil	Air	Paint	Cookware, pottery or glassware	Toys	Other
*Campylobacter* spp.	x	x	x	x	x	na	na	na	na	x
Non-typhoidal *Salmonella* spp.	x	x	x	x	x	na	na	na	na	x
Shiga toxin-producing *E*. *coli*	x	x	x	x	x	na	na	na	na	x
*Brucella* spp.	x	x	na	x	x	na	na	na	na	x
*Shigella* spp.	x	na	x	x	x	na	na	na	na	x
Enteropathogenic *E*. *coli*	x	x	x	x	na	na	na	na	na	x
Enterotoxigenic *E*. *coli*	x	x	x	x	na	na	na	na	na	x
*Cryptosporidium* spp.	x	x	x	x	na	na	na	na	na	x
*Giardia* spp.	x	x	x	x	na	na	na	na	na	x
*Salmonella* Typhi	x	na	x	x	na	na	na	na	na	x
*Vibrio cholerae*	x	na	x	x	na	na	na	na	na	x
*Entamoeba histolytica*	x	na	x	x	na	na	na	na	na	x
Norovirus	x	na	x	x	na	na	na	na	na	x
Hepatitis A virus	x	na	x	x	na	na	na	na	na	x
*Toxoplasma gondii*	x	x	na	x	x	na	na	na	na	x
*Echinococcus granulosus*	x	x	na	x	x	x	na	na	na	x
*Echinococcus multilocularis*	x	x	na	x	x	x	na	na	na	x
*Ascaris* spp.	x	x	x	x	x	na	na	na	na	x
Lead	x	na	na	x	x	x	x	x	x	x

Experts were asked to complete a set of tables for each assigned hazard and subregion. They were provided with these tables at the end of the telephone interview during which the seed questions were asked, and the facilitator went through several target questions with the experts to ensure that they understood the task. For the target items (but not the seed questions), the experts were free to consult any information sources they felt appropriate in the four-week period they were given to return the target item spreadsheets.

As with the seed questions, the experts were asked to provide their 5^th^, 50^th^ and 95^th^ percentile values for each question. Technically, the median values of a joint distribution do not need to add up to 100%. It is the associated mean values that should respect this criterion. However, the Classical Model operates on the basis of probability quantiles and experts are asked to give median values as central tendency indicators. Because we included a category ‘Other’, we thus asked about a joint distribution that logically spanned all possible exposure routes and—to be coherent therefore—the experts’ median values for source attribution percentages for a hazard should, in total, produce a value that does not grossly deviate from about 100%. In individual cases, where these sums were found to differ significantly from 100% (i.e. outside 100% +/- 10%), the experts concerned were asked to review their responses.

### Data analysis

Weights for individual experts were computed under the Classical Model formulation using the EXCALIBUR software by multiplying their calibration and informativeness scores, with the products then jointly normalized to sum to unity over all experts in the group. An expert was positively weighted only if his/her p-value was above a certain threshold, chosen to optimize the combined score across all seed items. In most applications of the Classical Model, a counterpart equally-weighted combination of experts’ distributions is also derived, typically for comparison with the corresponding performance-weighted solution. A detailed evaluation of the performance-based approach for this study has been described by Aspinall et al. [55]. See also the SI-1, Cooke [53], and Cooke et al. [56] for further details on the computation of expert performance weights.

The performance weighted attribution estimates were given in terms of the 5^th^, 50^th^, and 95^th^ % percentiles. A minimally informative density was fit to the uniform background measure which complies with these quantile constraints. First, for each exposure category within a hazard-subregion combination, independent vectors of 10,000 random deviates were generated from a Uniform(0, 100) distribution. The quantiles corresponding to these random deviates were then obtained via linear interpolation. As all possible exposure routes were included in the target questions (per definition by including an ‘Other’ option) and the exposure routes were considered mutual exclusive, it was necessary to ensure that the random attribution proportions summed to 100%. Therefore, a ‘normalization’ step was applied at each iteration, in which each random value was divided by the sum of random values for each exposure pathway. More precisely, if *f*_1_, …, *f*_*k*_ are relative frequencies which must sum to one, *f*_1_, …, *f*_*k*_ are first sampled independently, and then on each sample, *f*_*i*_ is replaced by $f_{i}/\sum_{j=1}^{k}f_{j}$. The 10,000 normalized random attribution proportions were then summarized by their median and a 95% uncertainty interval defined by the 2.5^th^ and 97.5^th^ percentiles. The resulting joint distributions (i.e., attributable proportion of illness per pathway, subregion and hazard) satisfy the ‘sum to 100%’ constraint which is closest to the product of the margins [57]. Data simulations were performed in R 3.1.1 [58] using functions available in the 'FERG' package [47].These distributions were applied by the hazard-specific Task Forces to estimate the burden of disease through the foodborne exposure route by multiplying the vectors of randomly-selected values for these parameters with a vector of randomly-selected values for the proportion that is foodborne, as described by Devleesshauwer et al. [47] and presented by Havelaar et al. [48].

## Results

A total of 299 potential experts were asked by email of their interest to participate in the study. Of these154 replied positively and they were requested to forward their CV, a filled in expert sheet and a signed declaration of interest. A total of 103 did that. Of these, 3 were not included due to lack of experience (n = 1) or possible conflicts of interest (n = 2). Of the 100 experts enrolled, 78 completed interviews with facilitators and 73 returned their spreadsheets with their responses to the target questions and seed questions. The single main reason for not completing the interview and returning the spreadsheet was time constraints. All responses were reviewed (i.e. checked for missing estimates, that sums across pathways were close to 100%, and that the 5^th^ percentile < 50^th^ percentile < 95^th^ percentiles, etc.) and some experts were contacted for clarification of the responses they had provided. One expert was dropped after not responding to requests for clarification. This resulted in the responses of 72 experts being included in the final dataset. Table 4 shows the distribution of experts across panels, and Fig 1 shows distribution of the experts by their geographical areas of expertise.

**Table 4:** The number of experts enrolled, interviewed and finally included in the elicitation across panels.

Hazard groups		Experts enrolled	Experts interviewed	Returned answers
**Diarrheal disease**				
Bacterial (incl. *S*. Typhi) pathogens and Norovirus	Subregional^a^	49	37	37
Intestinal protozoa	Global	12	9	9
**Other infectious disease**				
*Brucella* spp.	Global	10	8	7
Hepatitis A virus	Global	9	7	7
*Toxoplasma gondii*	Global	11	10	9
*Ascaris* spp.	Global	8	6	7
*Echinococcus* spp.	Global	7	6	6
**Chemicals**				
Lead	Global	10	9	6
**Total**^**b**^		100	78	72

^a^ Due to the subregional structure of these panels, the number of experts varied between 10 and 15 depending on the hazard and subregion.

^b^ As several experts served on more panels, the number of experts per panel does not add up to the total number of individual experts included.

### Expert performance

Due to the design of the study, having nine sets of seed questions, with 14 subregions and with some experts able to provide judgments only for certain regions or for some hazards, in the end there were 112 distinct datasets of expert elicitations (i.e. datasets that differed in membership or seed questions); in many cases, datasets differed only with respect to a few expert members. Overall, performance weight and equal weight combinations showed acceptable statistical accuracy. Only in the case of the dataset considering lead was the p-value of the performance-based combination small enough to cast doubt on the usual criterion for statistical accuracy, with p = 0.045 (i.e. less than 0.05 criterion). If the panels were independent (which they are not due to expert overlap) and statistically accurate, we should expect six panels’ p-values to fall below 5%.

**Fig 2:**
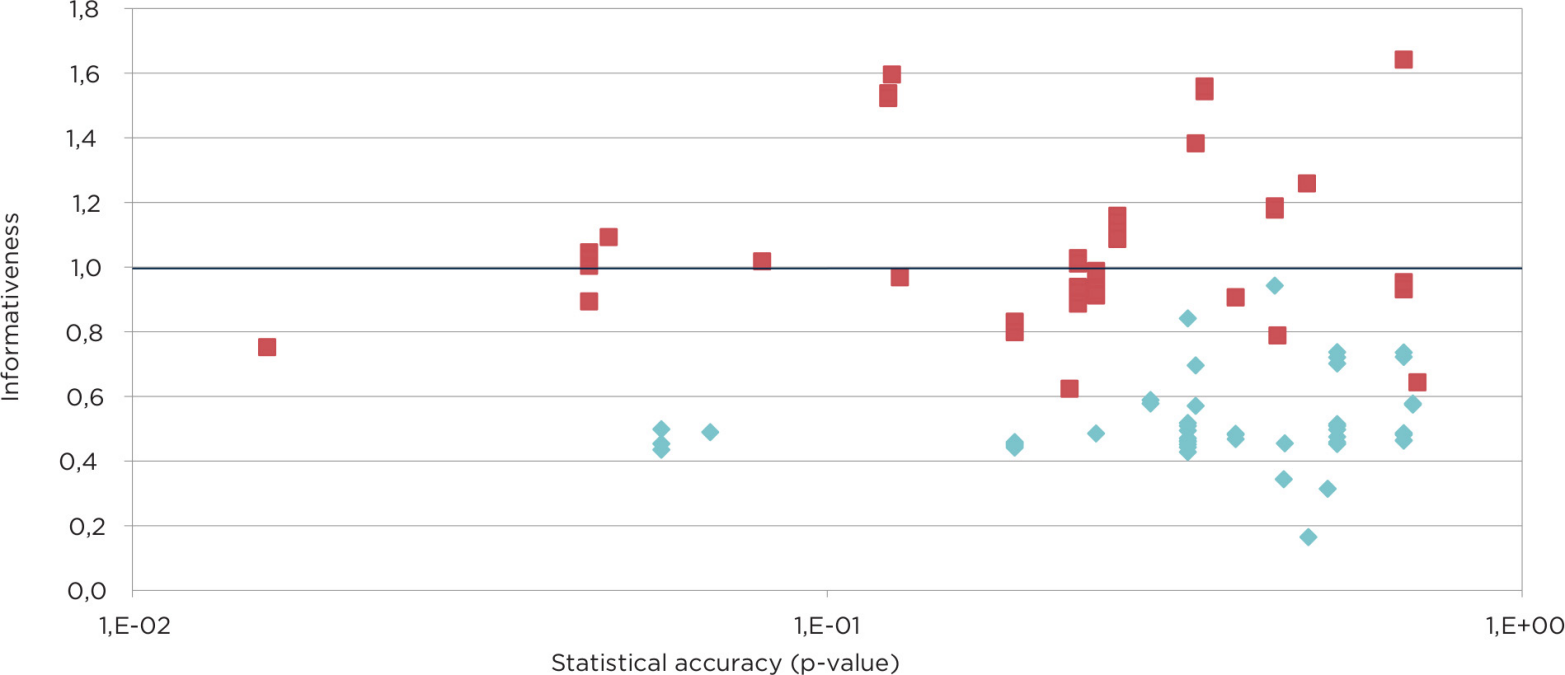
Statistical accuracy versus informativeness of the included experts when using equal weight (blue) or performance weight (red) combinations, respectively.

Results obtained by applying equal weights pooling and performance weights pooling were compared. The equal weights solutions tended to have higher statistical accuracy than those produced by applying the performance weights. In contrast, the informativeness properties of the equal weights solutions were much lower than those provided by performance weights solutions (Fig 2). This ‘trade-off’ between accuracy and informativeness when applying equal weights or performance weights is often seen, because the least accurate experts are typically the most informative, and their narrow 90% confidence bands often have little or no overlap. Moreover, the combined score using performance-based weights was above that of the equal weights pooling in 62% of the cases. It was, therefore, decided to use the performance weights combinations for constructing the joint probability distributions for the pathway attribution estimates as long as the statistical accuracy was acceptable. It should also be noted that the weight attributed to an expert–comprising the normalized product of his or her two scores–is dominated by the accuracy term, so that high informativeness cannot buy down poor accuracy. A unique feature of the present study is that a large number of experts were assessed using very similar variables, thereby allowing their informativeness scores to be compared. However, an in-depth analysis of the experts’ performance is outside the scope of the present paper, but is described elsewhere [55].

### Pathway attribution results

The collective results of the performance-based weighted expert responses are shown in Tables 5–9. We grouped the hazards according to the investigated transmission pathway to ease the presentation and discussion of results, following the order presented in Table 3. Individual expert responses can be found in (S3 File).

**Table 5:** Subregional estimates (median and 95% uncertainty interval) of the proportion of illnesses caused by *Campylobacter* spp., non-typhoidal *Salmonella* spp., Shiga toxin-producing *Escherichia coli* (STEC), *Brucella* spp. and *Shigella* spp. through each exposure pathway.

Hazard	Subregion	Food	Animal Contact (Domestic and Wild)	Human to Human Contact	Water	Soil	Other
*Campylobacter* spp.	AFR D	0.57 (0.31–0.77)	0.18 (0.00–0.42)	0.04 (0.00–0.22)	0.09 (0.01–0.29)	0.00 (0.00–0.12)	0.06 (0.00–0.16)
	AFR E	0.57 (0.29–0.77)	0.17 (0.00–0.42)	0.04 (0.00–0.23)	0.09 (0.00–0.30)	0.00 (0.00–0.12)	0.06 (0.00–0.16)
	AMR A	0.73 (0.38–0.91)	0.10 (0.00–0.37)	0.00 (0.00–0.20)	0.11 (0.00–0.32)	0.00 (0.00–0.11)	0.00 (0.00–0.02)
	AMR B	0.68 (0.41–0.82)	0.11 (0.00–0.33)	0.03 (0.00–0.21)	0.08 (0.00–0.27)	0.00 (0.00–0.11)	0.06 (0.00–0.16)
	AMR D	0.67 (0.37–0.81)	0.12 (0.01–0.36)	0.03 (0.00–0.21)	0.08 (0.00–0.29)	0.00 (0.00–0.15)	0.06 (0.00–0.16)
	EMR B	0.67 (0.38–0.82)	0.11 (0.01–0.35)	0.03 (0.00–0.27)	0.07 (0.00–0.29)	0.00 (0.00–0.15)	0.06 (0.00–0.15)
	EMR D	0.67 (0.41–0.82)	0.11 (0.00–0.34)	0.03 (0.00–0.22)	0.07 (0.00–0.27)	0.00 (0.00–0.20)	0.06 (0.00–0.15)
	EUR A	0.76 (0.44–0.93)	0.08 (0.00–0.31)	0.01 (0.00–0.13)	0.06 (0.00–0.35)	0.01 (0.00–0.09)	0.00 (0.00–0.08)
	EUR B	0.66 (0.34–0.87)	0.11 (0.00–0.39)	0.03 (0.00–0.21)	0.12 (0.00–0.40)	0.03 (0.00–0.13)	0.00 (0.00–0.05)
	EUR C	0.66 (0.34–0.87)	0.11 (0.00–0.38)	0.03 (0.00–0.23)	0.12 (0.00–0.39)	0.03 (0.00–0.19)	0.00 (0.00–0.02)
	SEAR B	0.57 (0.27–0.81)	0.13 (0.00–0.36)	0.11 (0.00–0.36)	0.05 (0.00–0.35)	0.03 (0.00–0.21)	0.02 (0.00–0.06)
	SEAR D	0.51 (0.03–0.79)	0.11 (0.00–0.39)	0.11 (0.01–0.41)	0.07 (0.00–0.44)	0.03 (0.00–0.32)	0.02 (0.00–0.10)
	WPR A	0.68 (0.40–0.89)	0.13 (0.00–0.33)	0.00 (0.00–0.23)	0.11 (0.00–0.32)	0.00 (0.00–0.08)	0.00 (0.00–0.01)
	WPR B	0.57 (0.25–0.82)	0.17 (0.00–0.42)	0.06 (0.00–0.34)	0.05 (0.00–0.32)	0.03 (0.00–0.15)	0.02 (0.00–0.07)
Non-typhoidal *Salmonella* spp.	AFR D	0.46 (0.13–0.74)	0.15 (0.00–0.43)	0.18 (0.00–0.48)	0.10 (0.00–0.39)	0.01 (0.00–0.13)	0.02 (0.00–0.06)
	AFR E	0.46 (0.10–0.73)	0.15 (0.00–0.42)	0.18 (0.00–0.48)	0.10 (0.00–0.40)	0.01 (0.00–0.19)	0.02 (0.00–0.08)
	AMR A	0.73 (0.38–0.91)	0.10 (0.00–0.39)	0.05 (0.00–0.28)	0.02 (0.00–0.22)	0.00 (0.00–0.09)	0.00 (0.00–0.05)
	AMR B	0.49 (0.09–0.74)	0.19 (0.00–0.45)	0.15 (0.00–0.40)	0.09 (0.00–0.32)	0.01 (0.00–0.12)	0.02 (0.00–0.05)
	AMR D	0.50 (0.14–0.75)	0.19 (0.00–0.46)	0.15 (0.00–0.39)	0.09 (0.00–0.31)	0.01 (0.00–0.12)	0.02 (0.00–0.05)
	EMR B	0.50 (0.18–0.75)	0.15 (0.00–0.43)	0.15 (0.01–0.38)	0.12 (0.00–0.33)	0.01 (0.00–0.19)	0.02 (0.00–0.04)
	EMR D	0.50 (0.19–0.74)	0.15 (0.00–0.43)	0.15 (0.01–0.39)	0.12 (0.00–0.32)	0.01 (0.00–0.21)	0.02 (0.00–0.05)
	EUR A	0.76 (0.47–0.94)	0.05 (0.00–0.30)	0.06 (0.00–0.26)	0.03 (0.00–0.21)	0.00 (0.00–0.11)	0.00 (0.00–0.14)
	EUR B	0.62 (0.31–0.84)	0.10 (0.00–0.37)	0.11 (0.01–0.32)	0.07 (0.00–0.32)	0.02 (0.00–0.12)	0.00 (0.00–0.01)
	EUR C	0.62 (0.32–0.84)	0.10 (0.00–0.36)	0.10 (0.00–0.32)	0.07 (0.00–0.32)	0.02 (0.00–0.12)	0.00 (0.00–0.01)
	SEAR B	0.58 (0.23–0.84)	0.06 (0.00–0.32)	0.10 (0.00–0.38)	0.11 (0.00–0.40)	0.02 (0.00–0.20)	0.00 (0.00–0.03)
	SEAR D	0.54 (0.00–0.85)	0.06 (0.00–0.37)	0.10 (0.00–0.42)	0.15 (0.00–0.59)	0.02 (0.00–0.29)	0.00 (0.00–0.06)
	WPR A	0.74 (0.45–0.93)	0.09 (0.00–0.31)	0.04 (0.00–0.28)	0.01 (0.00–0.22)	0.00 (0.00–0.08)	0.00 (0.00–0.04)
	WPR B	0.57 (0.25–0.82)	0.10 (0.00–0.33)	0.12 (0.00–0.35)	0.08 (0.00–0.37)	0.02 (0.00–0.21)	0.00 (0.00–0.01)
Shiga toxin-producing *E*. *coli*	AFR D	0.42 (0.19–0.66)	0.21 (0.04–0.46)	0.16 (0.00–0.33)	0.10 (0.00–0.30)	0.05 (0.00–0.25)	0.00 (0.00–0.03)
	AFR E	0.43 (0.14–0.66)	0.21 (0.04–0.46)	0.17 (0.01–0.34)	0.10 (0.00–0.34)	0.05 (0.00–0.19)	0.00 (0.00–0.03)
	AMR A	0.59 (0.19–0.84)	0.13 (0.00–0.41)	0.07 (0.00–0.32)	0.07 (0.00–0.31)	0.00 (0.00–0.13)	0.00 (0.00–0.27)
	AMR B	0.53 (0.24–0.73)	0.17 (0.01–0.44)	0.11 (0.01–0.29)	0.08 (0.00–0.32)	0.04 (0.00–0.21)	0.00 (0.00–0.03)
	AMR D	0.53 (0.24–0.75)	0.15 (0.00–0.43)	0.11 (0.01–0.29)	0.09 (0.00–0.32)	0.04 (0.00–0.17)	0.00 (0.00–0.03)
	EMR B	0.53 (0.24–0.76)	0.15 (0.02–0.43)	0.11 (0.00–0.29)	0.10 (0.00–0.37)	0.04 (0.00–0.18)	0.00 (0.00–0.03)
	EMR D	0.52 (0.26–0.75)	0.14 (0.01–0.42)	0.11 (0.01–0.30)	0.10 (0.00–0.37)	0.04 (0.00–0.17)	0.00 (0.00–0.03)
	EUR A	0.60 (0.26–0.83)	0.11 (0.01–0.37)	0.08 (0.00–0.33)	0.07 (0.00–0.33)	0.03 (0.00–0.19)	0.00 (0.00–0.14)
	EUR B	0.49 (0.15–0.75)	0.12 (0.00–0.42)	0.10 (0.01–0.32)	0.09 (0.00–0.38)	0.08 (0.00–0.35)	0.00 (0.00–0.01)
	EUR C	0.49 (0.15–0.75)	0.12 (0.00–0.42)	0.10 (0.01–0.32)	0.09 (0.00–0.36)	0.08 (0.00–0.35)	0.00 (0.00–0.01)
	SEAR B	0.41 (0.10–0.70)	0.12 (0.00–0.47)	0.07 (0.00–0.31)	0.23 (0.00–0.53)	0.06 (0.00–0.26)	0.00 (0.00–0.01)
	SEAR D	0.40 (0.08–0.71)	0.13 (0.00–0.47)	0.06 (0.00–0.35)	0.23 (0.00–0.53)	0.06 (0.00–0.26)	0.00 (0.00–0.02)
	WPR A	0.57 (0.25–0.82)	0.14 (0.00–0.36)	0.07 (0.00–0.35)	0.07 (0.00–0.29)	0.00 (0.00–0.16)	0.00 (0.00–0.24)
	WPR B	0.43 (0.12–0.73)	0.12 (0.00–0.44)	0.07 (0.00–0.35)	0.22 (0.00–0.46)	0.06 (0.00–0.27)	0.00 (0.00–0.01)
*Brucella* spp.	AFR D	0.44 (0.10–0.68)	0.50 (0.26–0.81)	na	0.01 (0.00–0.08)	0.01 (0.00–0.10)	0.01 (0.00–0.06)
	AFR E	0.44 (0.06–0.70)	0.50 (0.22–0.83)	na	0.01 (0.00–0.12)	0.01 (0.00–0.11)	0.01 (0.00–0.06)
	AMR A	0.75 (0.28–0.93)	0.19 (0.00–0.62)	na	0.01 (0.00–0.04)	0.01 (0.00–0.09)	0.01 (0.00–0.12)
	AMR B	0.44 (0.09–0.69)	0.50 (0.24–0.81)	na	0.01 (0.00–0.08)	0.01 (0.00–0.12)	0.01 (0.00–0.08)
	AMR D	0.44 (0.09–0.72)	0.50 (0.18–0.81)	na	0.01 (0.00–0.12)	0.01 (0.00–0.11)	0.01 (0.00–0.06)
	EMR B	0.51 (0.08–0.80)	0.43 (0.11–0.81)	na	0.01 (0.00–0.07)	0.01 (0.00–0.08)	0.01 (0.00–0.11)
	EMR D	0.44 (0.07–0.70)	0.50 (0.20–0.83)	na	0.01 (0.00–0.14)	0.01 (0.00–0.15)	0.01 (0.00–0.06)
	EUR A	0.66 (0.23–0.90)	0.23 (0.01–0.60)	na	0.01 (0.00–0.04)	0.01 (0.00–0.05)	0.02 (0.00–0.35)
	EUR B	0.45 (0.09–0.71)	0.50 (0.20–0.81)	na	0.01 (0.00–0.07)	0.01 (0.00–0.08)	0.01 (0.00–0.06)
	EUR C	0.44 (0.10–0.73)	0.50 (0.18–0.81)	na	0.01 (0.00–0.06)	0.01 (0.00–0.06)	0.01 (0.00–0.06)
	SEAR B	0.51 (0.07–0.81)	0.43 (0.10–0.81)	na	0.01 (0.00–0.07)	0.01 (0.00–0.07)	0.01 (0.00–0.07)
	SEAR D	0.45 (0.07–0.70)	0.50 (0.22–0.82)	na	0.01 (0.00–0.08)	0.01 (0.00–0.07)	0.01 (0.00–0.06)
	WPR A	0.71 (0.28–0.92)	0.18 (0.00–0.58)	na	0.01 (0.00–0.09)	0.01 (0.00–0.26)	0.02 (0.00–0.30)
	WPR B	0.51 (0.07–0.80)	0.43 (0.12–0.81)	na	0.01 (0.00–0.07)	0.01 (0.00–0.07)	0.01 (0.00–0.07)
*Shigella* spp.	AFR D	0.15 (0.00–0.52)	na	0.50 (0.06–0.81)	0.27 (0.03–0.62)	0.00 (0.00–0.19)	0.00 (0.00–0.13)
	AFR E	0.15 (0.00–0.51)	na	0.50 (0.08–0.80)	0.26 (0.05–0.61)	0.00 (0.00–0.19)	0.00 (0.00–0.16)
	AMR A	0.12 (0.00–0.46)	na	0.69 (0.33–0.93)	0.10 (0.00–0.41)	0.00 (0.00–0.21)	0.00 (0.00–0.06)
	AMR B	0.14 (0.00–0.52)	na	0.51 (0.10–0.81)	0.27 (0.03–0.61)	0.00 (0.00–0.18)	0.00 (0.00–0.06)
	AMR D	0.14 (0.00–0.52)	na	0.51 (0.11–0.80)	0.27 (0.02–0.60)	0.00 (0.00–0.20)	0.00 (0.00–0.02)
	EMR B	0.14 (0.00–0.52)	na	0.51 (0.11–0.81)	0.28 (0.03–0.61)	0.00 (0.00–0.17)	0.00 (0.00–0.02)
	EMR D	0.14 (0.00–0.52)	na	0.51 (0.11–0.81)	0.28 (0.02–0.61)	0.00 (0.00–0.18)	0.00 (0.00–0.02)
	EUR A	0.07 (0.00–0.46)	na	0.54 (0.14–0.90)	0.12 (0.00–0.52)	0.01 (0.00–0.20)	0.00 (0.00–0.55)
	EUR B	0.11 (0.00–0.50)	na	0.44 (0.10–0.75)	0.31 (0.04–0.60)	0.02 (0.00–0.20)	0.02 (0.00–0.21)
	EUR C	0.19 (0.00–0.51)	na	0.43 (0.07–0.70)	0.26 (0.02–0.53)	0.01 (0.00–0.20)	0.05 (0.00–0.22)
	SEAR B	0.36 (0.01–0.68)	na	0.30 (0.01–0.65)	0.26 (0.01–0.59)	0.04 (0.00–0.21)	0.01 (0.00–0.03)
	SEAR D	0.34 (0.01–0.69)	na	0.25 (0.00–0.64)	0.29 (0.01–0.65)	0.04 (0.00–0.26)	0.01 (0.00–0.06)
	WPR A	0.13 (0.00–0.50)	na	0.66 (0.25–0.91)	0.12 (0.00–0.42)	0.00 (0.00–0.22)	0.00 (0.00–0.19)
	WPR B	0.36 (0.01–0.70)	na	0.28 (0.00–0.65)	0.27 (0.01–0.60)	0.04 (0.00–0.22)	0.01 (0.00–0.03)

**Table 6:** Subregional estimates (median and 95% uncertainty interval) of the proportion of illnesses caused by enteropathogenic *E*. (EPEC), enterotoxigenic *E*. *coli* (ETEC), *Cryptosporidium* spp. and *Giardia* spp. through each exposure pathway.

Hazard	Subregion	Food	Animal Contact (Domestic and Wild)	Human to Human Contact	Water	Other
Enteropathogenic *E*. *Coli*	AFR D	0.29 (0.02–0.62)	0.00 (0.00–0.33)	0.16 (0.00–0.51)	0.45 (0.12–0.76)	0.00 (0.00–0.01)
	AFR E	0.29 (0.01–0.62)	0.00 (0.00–0.32)	0.16 (0.00–0.51)	0.46 (0.10–0.76)	0.00 (0.00–0.01)
	AMR A	0.72 (0.20–0.97)	0.00 (0.00–0.31)	0.11 (0.00–0.53)	0.00 (0.00–0.57)	0.00 (0.00–0.01)
	AMR B	0.29 (0.01–0.62)	0.00 (0.00–0.34)	0.16 (0.00–0.50)	0.46 (0.12–0.76)	0.00 (0.00–0.01)
	AMR D	0.30 (0.03–0.61)	0.00 (0.00–0.33)	0.15 (0.00–0.47)	0.47 (0.13–0.74)	0.00 (0.00–0.01)
	EMR B	0.31 (0.06–0.62)	0.00 (0.00–0.35)	0.14 (0.00–0.44)	0.46 (0.11–0.70)	0.00 (0.00–0.01)
	EMR D	0.31 (0.05–0.62)	0.00 (0.00–0.37)	0.14 (0.00–0.44)	0.45 (0.10–0.70)	0.00 (0.00–0.01)
	EUR A	0.64 (0.17–0.90)	0.05 (0.00–0.38)	0.17 (0.00–0.58)	0.03 (0.00–0.31)	0.00 (0.00–0.21)
	EUR B	0.48 (0.06–0.81)	0.08 (0.00–0.41)	0.26 (0.00–0.65)	0.08 (0.00–0.43)	0.00 (0.00–0.01)
	EUR C	0.48 (0.06–0.81)	0.09 (0.00–0.42)	0.26 (0.00–0.65)	0.08 (0.00–0.42)	0.00 (0.00–0.02)
	SEAR B	0.29 (0.01–0.62)	0.09 (0.00–0.34)	0.29 (0.01–0.62)	0.27 (0.01–0.58)	0.00 (0.00–0.02)
	SEAR D	0.29 (0.01–0.67)	0.09 (0.00–0.38)	0.27 (0.00–0.65)	0.27 (0.00–0.63)	0.00 (0.00–0.05)
	WPR A	0.69 (0.16–0.94)	0.00 (0.00–0.34)	0.18 (0.00–0.66)	0.00 (0.00–0.30)	0.00 (0.00–0.02)
	WPR B	0.30 (0.01–0.62)	0.14 (0.00–0.40)	0.23 (0.00–0.59)	0.26 (0.02–0.55)	0.00 (0.00–0.01)
Enterotoxigenic *E*. *coli*	AFR D	0.33 (0.09–0.65)	0.00 (0.00–0.33)	0.13 (0.00–0.44)	0.45 (0.12–0.71)	0.00 (0.00–0.01)
	AFR E	0.33 (0.06–0.64)	0.00 (0.00–0.33)	0.13 (0.00–0.45)	0.45 (0.09–0.71)	0.00 (0.00–0.01)
	AMR A	0.36 (0.12–0.63)	0.04 (0.00–0.32)	0.15 (0.00–0.37)	0.42 (0.11–0.66)	0.00 (0.00–0.19)
	AMR B	0.34 (0.08–0.65)	0.00 (0.00–0.34)	0.12 (0.00–0.42)	0.46 (0.11–0.70)	0.00 (0.00–0.13)
	AMR D	0.36 (0.07–0.68)	0.00 (0.00–0.32)	0.13 (0.00–0.43)	0.47 (0.10–0.72)	0.00 (0.00–0.01)
	EMR B	0.34 (0.07–0.65)	0.00 (0.00–0.31)	0.13 (0.00–0.42)	0.49 (0.10–0.72)	0.00 (0.00–0.01)
	EMR D	0.35 (0.05–0.66)	0.00 (0.00–0.31)	0.12 (0.00–0.41)	0.48 (0.12–0.73)	0.00 (0.00–0.01)
	EUR A	0.42 (0.09–0.73)	0.05 (0.00–0.31)	0.26 (0.01–0.60)	0.18 (0.00–0.53)	0.00 (0.00–0.08)
	EUR B	0.43 (0.05–0.73)	0.05 (0.00–0.34)	0.31 (0.02–0.66)	0.14 (0.00–0.47)	0.00 (0.00–0.18)
	EUR C	0.43 (0.06–0.72)	0.05 (0.00–0.34)	0.31 (0.02–0.66)	0.14 (0.00–0.47)	0.00 (0.00–0.20)
	SEAR B	0.38 (0.03–0.73)	0.05 (0.00–0.32)	0.09 (0.00–0.51)	0.39 (0.02–0.71)	0.00 (0.00–0.02)
	SEAR D	0.37 (0.02–0.73)	0.06 (0.00–0.34)	0.09 (0.00–0.52)	0.38 (0.03–0.73)	0.00 (0.00–0.11)
	WPR A	0.38 (0.10–0.72)	0.04 (0.00–0.29)	0.20 (0.00–0.53)	0.33 (0.00–0.61)	0.00 (0.00–0.01)
	WPR B	0.38 (0.03–0.72)	0.04 (0.00–0.29)	0.08 (0.00–0.50)	0.39 (0.04–0.71)	0.00 (0.00–0.20)
*Cryptosporidium* spp.	AFR D	0.15 (0.00–0.44)	0.06 (0.00–0.27)	0.38 (0.01–0.72)	0.35 (0.01–0.68)	0.01 (0.00–0.16)
	AFR E	0.15 (0.00–0.47)	0.05 (0.00–0.26)	0.36 (0.01–0.72)	0.37 (0.01–0.71)	0.01 (0.00–0.17)
	AMR A	0.16 (0.01–0.44)	0.10 (0.01–0.42)	0.30 (0.03–0.64)	0.37 (0.08–0.72)	0.00 (0.00–0.09)
	AMR B	0.11 (0.01–0.38)	0.20 (0.02–0.47)	0.35 (0.07–0.66)	0.26 (0.05–0.61)	0.00 (0.00–0.09)
	AMR D	0.16 (0.01–0.44)	0.21 (0.03–0.49)	0.34 (0.07–0.66)	0.20 (0.03–0.59)	0.00 (0.00–0.08)
	EMR B	0.09 (0.00–0.41)	0.14 (0.00–0.46)	0.31 (0.02–0.65)	0.36 (0.05–0.69)	0.01 (0.00–0.17)
	EMR D	0.08 (0.00–0.36)	0.13 (0.00–0.43)	0.32 (0.01–0.66)	0.38 (0.06–0.71)	0.01 (0.00–0.17)
	EUR A	0.10 (0.00–0.39)	0.14 (0.00–0.44)	0.30 (0.01–0.65)	0.38 (0.03–0.70)	0.01 (0.00–0.09)
	EUR B	0.11 (0.00–0.39)	0.16 (0.00–0.46)	0.28 (0.01–0.64)	0.37 (0.02–0.68)	0.01 (0.00–0.08)
	EUR C	0.09 (0.00–0.40)	0.15 (0.00–0.48)	0.29 (0.01–0.64)	0.36 (0.05–0.70)	0.01 (0.00–0.09)
	SEAR B	0.10 (0.00–0.37)	0.13 (0.00–0.46)	0.31 (0.01–0.66)	0.38 (0.02–0.71)	0.01 (0.00–0.09)
	SEAR D	0.10 (0.00–0.42)	0.13 (0.00–0.46)	0.30 (0.01–0.66)	0.37 (0.03–0.71)	0.01 (0.00–0.15)
	WPR A	0.10 (0.00–0.40)	0.12 (0.00–0.46)	0.29 (0.01–0.66)	0.39 (0.03–0.72)	0.01 (0.00–0.09)
	WPR B	0.10 (0.00–0.45)	0.10 (0.00–0.45)	0.29 (0.01–0.66)	0.39 (0.04–0.73)	0.01 (0.00–0.10)
*Giardia* spp.	AFR D	0.11 (0.00–0.43)	0.03 (0.00–0.27)	0.43 (0.01–0.75)	0.33 (0.05–0.69)	0.02 (0.00–0.18)
	AFR E	0.11 (0.00–0.43)	0.03 (0.00–0.25)	0.44 (0.04–0.75)	0.32 (0.04–0.67)	0.02 (0.00–0.19)
	AMR A	0.11 (0.00–0.39)	0.14 (0.00–0.41)	0.25 (0.00–0.64)	0.42 (0.05–0.75)	0.00 (0.00–0.12)
	AMR B	0.12 (0.00–0.42)	0.18 (0.00–0.47)	0.32 (0.01–0.67)	0.30 (0.04–0.65)	0.00 (0.00–0.09)
	AMR D	0.12 (0.00–0.42)	0.18 (0.00–0.46)	0.36 (0.01–0.69)	0.26 (0.03–0.63)	0.00 (0.00–0.10)
	EMR B	0.13 (0.00–0.50)	0.02 (0.00–0.15)	0.45 (0.03–0.77)	0.32 (0.03–0.71)	0.01 (0.00–0.19)
	EMR D	0.13 (0.00–0.47)	0.02 (0.00–0.25)	0.39 (0.02–0.73)	0.35 (0.03–0.71)	0.01 (0.00–0.18)
	EUR A	0.11 (0.00–0.44)	0.02 (0.00–0.15)	0.47 (0.02–0.79)	0.32 (0.03–0.72)	0.01 (0.00–0.14)
	EUR B	0.12 (0.00–0.47)	0.02 (0.00–0.15)	0.44 (0.02–0.77)	0.34 (0.02–0.73)	0.01 (0.00–0.12)
	EUR C	0.12 (0.00–0.48)	0.02 (0.00–0.15)	0.44 (0.02–0.77)	0.34 (0.04–0.74)	0.01 (0.00–0.13)
	SEAR B	0.13 (0.00–0.48)	0.02 (0.00–0.23)	0.41 (0.02–0.74)	0.35 (0.02–0.72)	0.01 (0.00–0.17)
	SEAR D	0.13 (0.00–0.48)	0.02 (0.00–0.22)	0.41 (0.02–0.76)	0.35 (0.03–0.72)	0.01 (0.00–0.16)
	WPR A	0.12 (0.00–0.45)	0.02 (0.00–0.31)	0.46 (0.02–0.78)	0.29 (0.01–0.68)	0.01 (0.00–0.18)
	WPR B	0.14 (0.00–0.49)	0.02 (0.00–0.29)	0.43 (0.02–0.75)	0.30 (0.03–0.69)	0.01 (0.00–0.19)

**Table 7:** Subregional estimates (median and 95% uncertainty interval) of the proportion of illnesses caused by *Salmonella* Typhi, *Vibrio cholerae*, *Entamoeba histolytica*, Norovirus, and Hepatitis A virus through each exposure pathway.

Hazard	Subregion	Food	Human to Human Contact	Water	Other
*Salmonella* Typhi	AFR D	0.24 (0.00–0.58)	0.22 (0.00–0.54)	0.51 (0.13–0.82)	0.00 (0.00–0.09)
	AFR E	0.24 (0.00–0.58)	0.22 (0.00–0.53)	0.51 (0.16–0.81)	0.00 (0.00–0.10)
	AMR A	0.26 (0.00–0.64)	0.11 (0.00–0.48)	0.57 (0.14–0.87)	0.00 (0.00–0.37)
	AMR B	0.23 (0.00–0.59)	0.21 (0.00–0.53)	0.52 (0.14–0.82)	0.00 (0.00–0.10)
	AMR D	0.23 (0.00–0.56)	0.21 (0.00–0.52)	0.53 (0.18–0.81)	0.00 (0.00–0.09)
	EMR B	0.24 (0.00–0.58)	0.21 (0.00–0.53)	0.52 (0.15–0.82)	0.00 (0.00–0.10)
	EMR D	0.24 (0.00–0.58)	0.21 (0.00–0.53)	0.52 (0.15–0.83)	0.00 (0.00–0.10)
	EUR A	0.10 (0.00–0.53)	0.23 (0.00–0.72)	0.41 (0.00–0.83)	0.01 (0.00–0.66)
	EUR B	0.08 (0.00–0.43)	0.47 (0.16–0.78)	0.35 (0.04–0.62)	0.02 (0.00–0.21)
	EUR C	0.08 (0.00–0.43)	0.47 (0.15–0.78)	0.35 (0.03–0.62)	0.02 (0.00–0.21)
	SEAR B	0.43 (0.11–0.82)	0.12 (0.00–0.49)	0.40 (0.01–0.70)	0.00 (0.00–0.03)
	SEAR D	0.40 (0.01–0.81)	0.13 (0.00–0.54)	0.42 (0.00–0.80)	0.00 (0.00–0.10)
	WPR A	0.33 (0.00–0.84)	0.11 (0.00–0.55)	0.48 (0.00–0.86)	0.00 (0.00–0.36)
	WPR B	0.49 (0.10–0.84)	0.13 (0.00–0.51)	0.33 (0.01–0.66)	0.00 (0.00–0.03)
*Vibrio cholerae*	AFR D	0.21 (0.01–0.57)	0.02 (0.00–0.31)	0.72 (0.29–0.94)	0.00 (0.00–0.03)
	AFR E	0.21 (0.01–0.56)	0.02 (0.00–0.30)	0.72 (0.33–0.94)	0.00 (0.00–0.04)
	AMR A	0.30 (0.01–0.95)	0.02 (0.00–0.43)	0.59 (0.00–0.93)	0.00 (0.00–0.37)
	AMR B	0.25 (0.00–0.58)	0.02 (0.00–0.27)	0.70 (0.33–0.95)	0.00 (0.00–0.34)
	AMR D	0.25 (0.00–0.57)	0.02 (0.00–0.29)	0.69 (0.34–0.94)	0.00 (0.00–0.29)
	EMR B	0.23 (0.01–0.64)	0.02 (0.00–0.30)	0.69 (0.25–0.94)	0.00 (0.00–0.03)
	EMR D	0.23 (0.01–0.65)	0.02 (0.00–0.31)	0.70 (0.23–0.94)	0.00 (0.00–0.03)
	EUR A	0.31 (0.00–0.85)	0.03 (0.00–0.44)	0.44 (0.00–0.86)	0.01 (0.00–0.57)
	EUR B	0.46 (0.01–0.86)	0.11 (0.00–0.47)	0.36 (0.00–0.77)	0.00 (0.00–0.36)
	EUR C	0.46 (0.02–0.86)	0.11 (0.00–0.47)	0.36 (0.00–0.76)	0.00 (0.00–0.38)
	SEAR B	0.36 (0.04–0.78)	0.14 (0.00–0.50)	0.45 (0.02–0.79)	0.00 (0.00–0.02)
	SEAR D	0.25 (0.00–0.75)	0.08 (0.00–0.50)	0.58 (0.04–0.91)	0.00 (0.00–0.02)
	WPR A	0.25 (0.01–0.92)	0.04 (0.00–0.64)	0.56 (0.00–0.93)	0.00 (0.00–0.05)
	WPR B	0.29 (0.01–0.74)	0.13 (0.00–0.49)	0.51 (0.04–0.83)	0.00 (0.00–0.30)
**Norovirus	AFR D	0.15 (0.01–0.40)	0.68 (0.37–0.89)	0.07 (0.00–0.38)	0.04 (0.00–0.23)
	AFR E	0.15 (0.00–0.40)	0.68 (0.38–0.89)	0.07 (0.00–0.37)	0.04 (0.00–0.24)
	AMR A	0.23 (0.04–0.50)	0.50 (0.18–0.79)	0.22 (0.00–0.49)	0.00 (0.00–0.22)
	AMR B	0.14 (0.00–0.42)	0.72 (0.36–0.90)	0.06 (0.00–0.40)	0.04 (0.00–0.24)
	AMR D	0.15 (0.00–0.46)	0.72 (0.36–0.89)	0.06 (0.00–0.41)	0.04 (0.00–0.23)
	EMR B	0.15 (0.00–0.40)	0.72 (0.43–0.89)	0.07 (0.00–0.30)	0.04 (0.00–0.22)
	EMR D	0.15 (0.00–0.40)	0.72 (0.42–0.89)	0.06 (0.00–0.32)	0.04 (0.00–0.23)
	EUR A	0.26 (0.00–0.73)	0.43 (0.00–0.83)	0.17 (0.00–0.58)	0.00 (0.00–0.36)
	EUR B	0.23 (0.01–0.57)	0.32 (0.02–0.67)	0.33 (0.00–0.65)	0.04 (0.00–0.34)
	EUR C	0.23 (0.01–0.57)	0.33 (0.02–0.67)	0.33 (0.01–0.63)	0.04 (0.00–0.33)
	SEAR B	0.12 (0.00–0.48)	0.53 (0.13–0.83)	0.21 (0.00–0.53)	0.00 (0.00–0.42)
	SEAR D	0.15 (0.00–0.55)	0.46 (0.00–0.79)	0.29 (0.00–0.72)	0.00 (0.00–0.35)
	WPR A	0.22 (0.01–0.52)	0.48 (0.12–0.77)	0.22 (0.00–0.51)	0.00 (0.00–0.32)
	WPR B	0.15 (0.00–0.55)	0.46 (0.00–0.79)	0.28 (0.01–0.68)	0.00 (0.00–0.34)
**Hepatitis A virus	AFR D	0.36 (0.07–0.63)	0.40 (0.10–0.68)	0.17 (0.00–0.49)	0.04 (0.00–0.10)
	AFR E	0.29 (0.07–0.57)	0.36 (0.08–0.64)	0.30 (0.06–0.59)	0.02 (0.00–0.06)
	AMR A	0.42 (0.06–0.77)	0.46 (0.04–0.78)	0.01 (0.00–0.19)	0.10 (0.00–0.32)
	AMR B	0.31 (0.03–0.60)	0.46 (0.16–0.74)	0.11 (0.00–0.39)	0.09 (0.00–0.21)
	AMR D	0.32 (0.03–0.61)	0.35 (0.11–0.65)	0.26 (0.04–0.57)	0.04 (0.00–0.09)
	EMR B	0.35 (0.04–0.61)	0.42 (0.17–0.69)	0.15 (0.02–0.34)	0.09 (0.00–0.20)
	EMR D	0.32 (0.02–0.59)	0.36 (0.11–0.66)	0.22 (0.00–0.49)	0.08 (0.00–0.23)
	EUR A	0.42 (0.02–0.75)	0.46 (0.10–0.79)	0.01 (0.00–0.17)	0.10 (0.00–0.32)
	EUR B	0.35 (0.12–0.59)	0.35 (0.18–0.61)	0.20 (0.01–0.36)	0.08 (0.00–0.19)
	EUR C	0.34 (0.08–0.60)	0.42 (0.17–0.69)	0.14 (0.00–0.35)	0.09 (0.00–0.24)
	SEAR B	0.34 (0.05–0.60)	0.35 (0.14–0.65)	0.23 (0.04–0.55)	0.04 (0.00–0.09)
	SEAR D	0.29 (0.04–0.56)	0.37 (0.13–0.64)	0.29 (0.06–0.56)	0.02 (0.00–0.06)
	WPR A	0.42 (0.03–0.76)	0.46 (0.10–0.79)	0.01 (0.00–0.16)	0.10 (0.00–0.29)
	WPR B	0.34 (0.02–0.64)	0.36 (0.06–0.66)	0.21 (0.01–0.47)	0.08 (0.00–0.20)
*Entamoeba histolytica*	AFR D	0.30 (0.00–0.68)	0.37 (0.00–0.73)	0.25 (0.00–0.63)	0.04 (0.00–0.21)
	AFR E	0.30 (0.00–0.68)	0.37 (0.00–0.72)	0.24 (0.00–0.62)	0.04 (0.00–0.22)
	AMR A	0.25 (0.00–0.70)	0.34 (0.00–0.76)	0.33 (0.00–0.74)	0.00 (0.00–0.19)
	AMR B	0.21 (0.00–0.62)	0.38 (0.02–0.76)	0.32 (0.00–0.70)	0.00 (0.00–0.20)
	AMR D	0.17 (0.00–0.58)	0.37 (0.04–0.76)	0.37 (0.01–0.73)	0.00 (0.00–0.20)
	EMR B	0.24 (0.00–0.62)	0.42 (0.01–0.76)	0.24 (0.00–0.62)	0.04 (0.00–0.22)
	EMR D	0.28 (0.00–0.66)	0.39 (0.00–0.75)	0.25 (0.00–0.65)	0.04 (0.00–0.22)
	EUR A	0.33 (0.00–0.71)	0.49 (0.03–0.83)	0.15 (0.00–0.51)	0.01 (0.00–0.16)
	EUR B	0.30 (0.00–0.66)	0.42 (0.02–0.76)	0.20 (0.00–0.59)	0.04 (0.00–0.20)
	EUR C	0.26 (0.00–0.64)	0.42 (0.02–0.76)	0.23 (0.00–0.61)	0.04 (0.00–0.19)
	SEAR B	0.26 (0.00–0.65)	0.38 (0.00–0.75)	0.28 (0.00–0.68)	0.04 (0.00–0.18)
	SEAR D	0.25 (0.00–0.63)	0.37 (0.00–0.72)	0.29 (0.01–0.69)	0.04 (0.00–0.19)
	WPR A	0.25 (0.00–0.62)	0.41 (0.00–0.74)	0.26 (0.01–0.62)	0.04 (0.00–0.25)
	WPR B	0.27 (0.00–0.63)	0.41 (0.00–0.73)	0.24 (0.01–0.62)	0.05 (0.00–0.23)

**Table 8:** Subregional estimates (median and 95% uncertainty interval) of the proportion of illnesses caused by *Toxoplasma gondii*, *Echinococcus multilocularis*, *Echinococcus granulosus* and *Ascaris* spp. through each exposure pathway.

Hazard	Subregion	Food	Animal Contact (Domestic and Wild)	Human to Human Contact	Water	Soil	Air	Other
*Toxoplasma gondii*	AFR D	0.48 (0.24–0.76)	0.01 (0.00–0.20)	na	0.11 (0.00–0.37)	0.36 (0.07–0.57)	na	na
	AFR E	0.42 (0.20–0.70)	0.01 (0.00–0.19)	na	0.16 (0.02–0.41)	0.38 (0.05–0.58)	na	na
	AMR A	0.60 (0.30–0.81)	0.01 (0.00–0.28)	na	0.19 (0.01–0.42)	0.19 (0.00–0.46)	na	na
	AMR B	0.52 (0.27–0.77)	0.01 (0.00–0.20)	na	0.23 (0.01–0.45)	0.22 (0.00–0.46)	na	na
	AMR D	0.53 (0.27–0.77)	0.01 (0.00–0.21)	na	0.23 (0.02–0.44)	0.22 (0.00–0.45)	na	na
	EMR B	0.52 (0.27–0.80)	0.01 (0.00–0.20)	na	0.11 (0.01–0.29)	0.34 (0.02–0.56)	na	na
	EMR D	0.53 (0.29–0.77)	0.01 (0.00–0.20)	na	0.23 (0.02–0.43)	0.22 (0.00–0.42)	na	na
	EUR A	0.61 (0.35–0.82)	0.01 (0.00–0.21)	na	0.19 (0.02–0.36)	0.18 (0.00–0.40)	na	na
	EUR B	0.45 (0.23–0.76)	0.01 (0.00–0.20)	na	0.15 (0.02–0.35)	0.37 (0.01–0.58)	na	na
	EUR C	0.53 (0.31–0.78)	0.01 (0.00–0.20)	na	0.23 (0.03–0.41)	0.22 (0.01–0.41)	na	na
	SEAR B	0.52 (0.26–0.77)	0.01 (0.00–0.19)	na	0.23 (0.03–0.45)	0.22 (0.00–0.43)	na	na
	SEAR D	0.43 (0.09–0.73)	0.01 (0.00–0.22)	na	0.27 (0.03–0.58)	0.26 (0.00–0.56)	na	na
	WPR A	0.60 (0.33–0.81)	0.01 (0.00–0.21)	na	0.19 (0.02–0.37)	0.18 (0.00–0.43)	na	na
	WPR B	0.53 (0.29–0.77)	0.01 (0.00–0.20)	na	0.23 (0.04–0.43)	0.22 (0.00–0.43)	na	na
*Echinococcus granulosus*	AFR D	0.21 (0.07–0.42)	0.51 (0.25–0.72)	na	0.18 (0.01–0.34)	0.09 (0.00–0.20)	0.00 (0.00–0.06)	0.00 (0.00–0.01)
	AFR E	0.20 (0.05–0.40)	0.52 (0.27–0.73)	na	0.18 (0.00–0.35)	0.09 (0.00–0.19)	0.00 (0.00–0.06)	0.00 (0.00–0.06)
	AMR A	0.20 (0.03–0.40)	0.52 (0.30–0.75)	na	0.17 (0.00–0.31)	0.09 (0.00–0.20)	0.00 (0.00–0.14)	0.00 (0.00–0.01)
	AMR B	0.20 (0.02–0.43)	0.52 (0.28–0.73)	na	0.18 (0.00–0.34)	0.09 (0.00–0.22)	0.00 (0.00–0.14)	0.00 (0.00–0.01)
	AMR D	0.21 (0.05–0.41)	0.51 (0.29–0.72)	na	0.18 (0.01–0.35)	0.09 (0.00–0.23)	0.00 (0.00–0.13)	0.00 (0.00–0.01)
	EMR B	0.21 (0.05–0.43)	0.51 (0.28–0.73)	na	0.17 (0.00–0.32)	0.09 (0.00–0.19)	0.00 (0.00–0.14)	0.00 (0.00–0.06)
	EMR D	0.21 (0.06–0.41)	0.52 (0.28–0.72)	na	0.18 (0.00–0.32)	0.09 (0.00–0.18)	0.00 (0.00–0.14)	0.00 (0.00–0.01)
	EUR A	0.21 (0.04–0.40)	0.51 (0.29–0.72)	na	0.18 (0.00–0.33)	0.09 (0.00–0.20)	0.00 (0.00–0.14)	0.00 (0.00–0.01)
	EUR B	0.21 (0.06–0.40)	0.52 (0.27–0.73)	na	0.18 (0.00–0.33)	0.09 (0.00–0.19)	0.00 (0.00–0.15)	0.00 (0.00–0.01)
	EUR C	0.21 (0.04–0.40)	0.51 (0.26–0.73)	na	0.18 (0.00–0.35)	0.09 (0.00–0.21)	0.00 (0.00–0.15)	0.00 (0.00–0.01)
	SEAR B	0.21 (0.03–0.44)	0.51 (0.22–0.73)	na	0.18 (0.00–0.35)	0.09 (0.00–0.19)	0.00 (0.00–0.13)	0.00 (0.00–0.01)
	SEAR D	0.20 (0.06–0.40)	0.52 (0.29–0.73)	na	0.18 (0.00–0.34)	0.09 (0.00–0.19)	0.00 (0.00–0.14)	0.00 (0.00–0.01)
	WPR A	0.20 (0.01–0.39)	0.53 (0.30–0.75)	na	0.18 (0.00–0.33)	0.09 (0.00–0.20)	0.00 (0.00–0.13)	0.00 (0.00–0.01)
	WPR B	0.21 (0.05–0.43)	0.51 (0.29–0.73)	na	0.17 (0.00–0.32)	0.09 (0.00–0.21)	0.00 (0.00–0.14)	0.00 (0.00–0.01)
*Echinococcus multilocularis*	AFR D	0.58 (0.00–0.87)	0.02 (0.00–0.42)	na	0.20 (0.00–0.61)	0.20 (0.00–0.63)	0.00 (0.00–0.03)	0.00 (0.00–0.00)
	AFR E	0.58 (0.00–0.87)	0.02 (0.00–0.41)	na	0.20 (0.00–0.62)	0.20 (0.00–0.61)	0.00 (0.00–0.03)	0.00 (0.00–0.00)
	AMR A	0.51 (0.13–0.79)	0.03 (0.00–0.50)	na	0.17 (0.01–0.40)	0.16 (0.01–0.38)	0.00 (0.00–0.11)	0.00 (0.00–0.03)
	AMR B	0.58 (0.00–0.87)	0.02 (0.00–0.38)	na	0.20 (0.00–0.62)	0.20 (0.00–0.61)	0.00 (0.00–0.03)	0.00 (0.00–0.00)
	AMR D	0.58 (0.00–0.88)	0.02 (0.00–0.41)	na	0.19 (0.00–0.61)	0.20 (0.00–0.60)	0.00 (0.00–0.03)	0.00 (0.00–0.00)
	EMR B	0.43 (0.09–0.73)	0.14 (0.00–0.55)	na	0.17 (0.00–0.42)	0.17 (0.00–0.42)	0.00 (0.00–0.06)	0.00 (0.00–0.01)
	EMR D	0.48 (0.00–0.77)	0.12 (0.00–0.49)	na	0.20 (0.00–0.54)	0.20 (0.00–0.53)	0.00 (0.00–0.04)	0.00 (0.00–0.00)
	EUR A	0.52 (0.15–0.79)	0.03 (0.00–0.48)	na	0.17 (0.01–0.40)	0.16 (0.00–0.39)	0.00 (0.00–0.11)	0.00 (0.00–0.03)
	EUR B	0.45 (0.12–0.72)	0.13 (0.00–0.52)	na	0.18 (0.02–0.38)	0.17 (0.00–0.37)	0.00 (0.00–0.13)	0.00 (0.00–0.03)
	EUR C	0.44 (0.12–0.72)	0.14 (0.00–0.53)	na	0.17 (0.01–0.38)	0.17 (0.00–0.37)	0.00 (0.00–0.12)	0.00 (0.00–0.03)
	SEAR B	0.58 (0.00–0.88)	0.02 (0.00–0.41)	na	0.20 (0.00–0.61)	0.20 (0.00–0.61)	0.00 (0.00–0.03)	0.00 (0.00–0.00)
	SEAR D	0.58 (0.00–0.88)	0.02 (0.00–0.37)	na	0.20 (0.00–0.62)	0.20 (0.00–0.60)	0.00 (0.00–0.05)	0.00 (0.00–0.00)
	WPR A	0.51 (0.09–0.81)	0.04 (0.00–0.52)	na	0.16 (0.00–0.41)	0.16 (0.00–0.40)	0.00 (0.00–0.03)	0.00 (0.00–0.01)
	WPR B	0.48 (0.00–0.78)	0.12 (0.00–0.49)	na	0.20 (0.00–0.54)	0.20 (0.00–0.54)	0.00 (0.00–0.12)	0.00 (0.00–0.03)
*Ascaris* spp.	AFR D	0.38 (0.10–0.66)	0.00 (0.00–0.09)	0.00 (0.00–0.08)	0.19 (0.07–0.40)	0.39 (0.07–0.65)	na	0.00 (0.00–0.06)
	AFR E	0.38 (0.07–0.67)	0.00 (0.00–0.09)	0.00 (0.00–0.09)	0.19 (0.07–0.41)	0.39 (0.05–0.65)	na	0.00 (0.00–0.06)
	AMR A	0.83 (0.43–0.97)	0.00 (0.00–0.29)	0.00 (0.00–0.08)	0.05 (0.00–0.18)	0.06 (0.00–0.42)	na	0.00 (0.00–0.06)
	AMR B	0.55 (0.17–0.75)	0.00 (0.00–0.13)	0.00 (0.00–0.09)	0.19 (0.06–0.40)	0.22 (0.05–0.50)	na	0.00 (0.00–0.04)
	AMR D	0.37 (0.07–0.68)	0.00 (0.00–0.15)	0.00 (0.00–0.08)	0.18 (0.05–0.41)	0.41 (0.04–0.69)	na	0.00 (0.00–0.04)
	EMR B	0.55 (0.15–0.77)	0.00 (0.00–0.10)	0.00 (0.00–0.07)	0.20 (0.02–0.44)	0.22 (0.02–0.51)	na	0.00 (0.00–0.06)
	EMR D	0.55 (0.18–0.75)	0.00 (0.00–0.10)	0.00 (0.00–0.09)	0.20 (0.04–0.43)	0.21 (0.04–0.51)	na	0.00 (0.00–0.05)
	EUR A	0.85 (0.47–0.97)	0.00 (0.00–0.25)	0.00 (0.00–0.09)	0.05 (0.00–0.18)	0.06 (0.00–0.38)	na	0.00 (0.00–0.06)
	EUR B	0.55 (0.13–0.76)	0.00 (0.00–0.27)	0.00 (0.00–0.10)	0.19 (0.03–0.40)	0.22 (0.02–0.50)	na	0.00 (0.00–0.06)
	EUR C	0.55 (0.14–0.76)	0.00 (0.00–0.25)	0.00 (0.00–0.12)	0.19 (0.03–0.40)	0.22 (0.04–0.50)	na	0.00 (0.00–0.05)
	SEAR B	0.54 (0.18–0.75)	0.00 (0.00–0.14)	0.00 (0.00–0.08)	0.20 (0.03–0.44)	0.22 (0.01–0.52)	na	0.00 (0.00–0.05)
	SEAR D	0.39 (0.11–0.68)	0.00 (0.00–0.12)	0.00 (0.00–0.07)	0.20 (0.04–0.44)	0.38 (0.04–0.65)	na	0.00 (0.00–0.06)
	WPR A	0.85 (0.47–0.97)	0.00 (0.00–0.23)	0.00 (0.00–0.09)	0.05 (0.00–0.19)	0.06 (0.00–0.37)	na	0.00 (0.00–0.06)
	WPR B	0.54 (0.16–0.77)	0.00 (0.00–0.24)	0.00 (0.00–0.11)	0.20 (0.02–0.43)	0.21 (0.02–0.49)	na	0.00 (0.00–0.06)

**Table 9:** Subregional estimates (median and 95% uncertainty interval) of the proportion of illnesses caused by exposure to lead through each pathway.

Hazard	Subregion	Food	Water	Soil	Air	Paint	Cookware, pottery or glassware	Toys	Other
Lead	AFR D	0.17 (0.00–0.37)	0.22 (0.06–0.48)	0.12 (0.00–0.27)	0.20 (0.03–0.39)	0.08 (0.00–0.32)	0.09 (0.01–0.24)	0.04 (0.00–0.16)	0.00 (0.00–0.04)
	AFR E	0.17 (0.00–0.37)	0.28 (0.06–0.54)	0.10 (0.00–0.28)	0.18 (0.00–0.38)	0.08 (0.00–0.33)	0.07 (0.00–0.27)	0.02 (0.00–0.17)	0.00 (0.00–0.04)
	AMR A	0.24 (0.01–0.49)	0.30 (0.05–0.61)	0.09 (0.00–0.27)	0.12 (0.00–0.50)	0.04 (0.00–0.35)	0.05 (0.00–0.22)	0.05 (0.00–0.19)	0.00 (0.00–0.02)
	AMR B	0.19 (0.00–0.41)	0.22 (0.04–0.46)	0.04 (0.00–0.16)	0.26 (0.00–0.51)	0.06 (0.00–0.35)	0.09 (0.01–0.38)	0.02 (0.00–0.20)	0.00 (0.00–0.02)
	AMR D	0.17 (0.00–0.40)	0.14 (0.03–0.42)	0.13 (0.00–0.35)	0.29 (0.00–0.57)	0.05 (0.00–0.36)	0.04 (0.00–0.35)	0.02 (0.00–0.19)	0.00 (0.00–0.02)
	EMR B	0.19 (0.01–0.37)	0.21 (0.06–0.42)	0.10 (0.00–0.22)	0.21 (0.00–0.41)	0.09 (0.00–0.36)	0.07 (0.00–0.32)	0.02 (0.00–0.23)	0.00 (0.00–0.02)
	EMR D	0.11 (0.00–0.31)	0.09 (0.03–0.23)	0.07 (0.00–0.55)	0.38 (0.10–0.66)	0.04 (0.00–0.24)	0.06 (0.00–0.23)	0.02 (0.00–0.18)	0.00 (0.00–0.01)
	EUR A	0.23 (0.00–0.46)	0.19 (0.05–0.47)	0.10 (0.00–0.24)	0.16 (0.00–0.37)	0.14 (0.04–0.48)	0.05 (0.00–0.20)	0.02 (0.00–0.18)	0.00 (0.00–0.02)
	EUR B	0.23 (0.00–0.47)	0.16 (0.02–0.40)	0.12 (0.00–0.30)	0.18 (0.00–0.40)	0.05 (0.00–0.38)	0.09 (0.01–0.28)	0.06 (0.00–0.23)	0.00 (0.00–0.02)
	EUR C	0.19 (0.00–0.37)	0.29 (0.11–0.54)	0.11 (0.00–0.30)	0.12 (0.00–0.35)	0.03 (0.00–0.39)	0.06 (0.00–0.25)	0.04 (0.00–0.22)	0.00 (0.00–0.03)
	SEAR B	0.17 (0.00–0.40)	0.17 (0.02–0.38)	0.07 (0.00–0.23)	0.28 (0.00–0.54)	0.05 (0.00–0.36)	0.08 (0.00–0.33)	0.05 (0.00–0.24)	0.00 (0.00–0.01)
	SEAR D	0.21 (0.00–0.46)	0.15 (0.05–0.31)	0.11 (0.00–0.27)	0.24 (0.05–0.46)	0.06 (0.00–0.30)	0.11 (0.03–0.27)	0.03 (0.00–0.23)	0.00 (0.00–0.01)
	WPR A	0.12 (0.00–0.30)	0.14 (0.03–0.36)	0.14 (0.00–0.32)	0.27 (0.00–0.51)	0.09 (0.00–0.38)	0.11 (0.03–0.37)	0.03 (0.00–0.19)	0.00 (0.00–0.01)
	WPR B	0.12 (0.00–0.30)	0.22 (0.06–0.45)	0.06 (0.00–0.19)	0.30 (0.00–0.53)	0.08 (0.00–0.38)	0.09 (0.00–0.36)	0.03 (0.00–0.24)	0.00 (0.00–0.01)

Overall, and for most estimates, there is considerable uncertainty, reflecting: 1) variations in uncertainty estimations between individual experts; 2) that, for some hazards, the values provided by experts having high performance weights in the analysis did not accord with one another, and 3) that, for some subregions or hazards, the number of contributing experts was small (<7). Thus, the broad uncertainty intervals are most likely reflecting current shortcomings in hard scientific evidence about the relative contribution to human disease from each of the transmission pathways.

**Fig 3:**
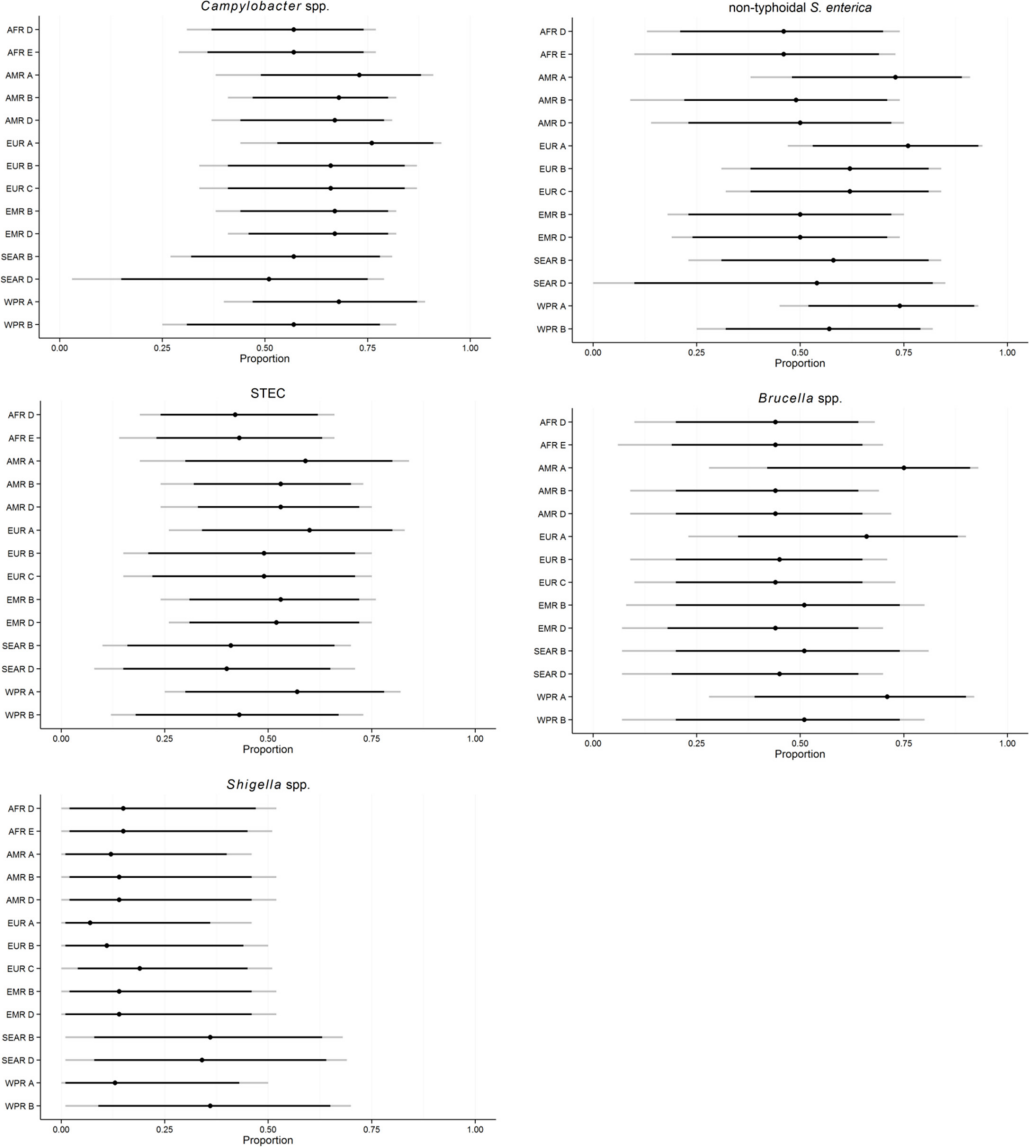
Subregional estimates of the proportion of foodborne illnesses caused by *Campylobacter* spp., non-typhoidal *Salmonella* spp., Shiga toxin-producing *Escherichia coli* (STEC), *Brucella* spp. and *Shigella* spp. Indicated on the line plot are the 2.5^th^, 5^th^, 50^th^, 95^th^ and 97,5^th^ percentiles.

Fig 3 shows the subregional estimates of the foodborne proportion for *Campylobacter* spp., non-typhoidal *Salmonella* spp., Shiga toxin-producing *Escherichia coli* (STEC), *Brucella* spp. and *Shigella* spp. For *Salmonella* spp. and *Brucella* spp., there is a clear pattern that the foodborne proportion is considered more important in the developed subregions (AMR A, EUR A and WPR A, see Fig 1 for subregions) compared to developing subregions. Although less distinct, this pattern can also be seen for *Campylobacter* spp. and STEC. For *Campylobacter* spp., *Salmonella* spp. and STEC the foodborne transmission route was assessed by the experts to be the most important route in all subregions, followed by direct animal contact, human-to-human transmission and waterborne transmission in varying order, but generally, with medians below 0.25 (Table 5). It is notable that in all subregions, over half of campylobacteriosis was deemed to be foodborne (Table 5). For *Brucella* spp., direct animal contact was considered equally or more important than foodborne transmission in developing subregions. Human-to-human transmission was considered the most important route for *Shigella* spp. in the majority of subregions. Proportion foodborne *Shigella* spp. infections ranged from 0.07 (95%CI: 0.00–0.46) in EUR A to 0.36 (95%CI: 0.01–0.70) in WPR A (Table 5). Overall, foodborne transmission was assessed to be more important in South-East Asian and Western Pacific subregions than in other parts of the world. Transmission through soil or other routes was recognised by the experts to be of minor importance for these five pathogens.

**Fig 4:**
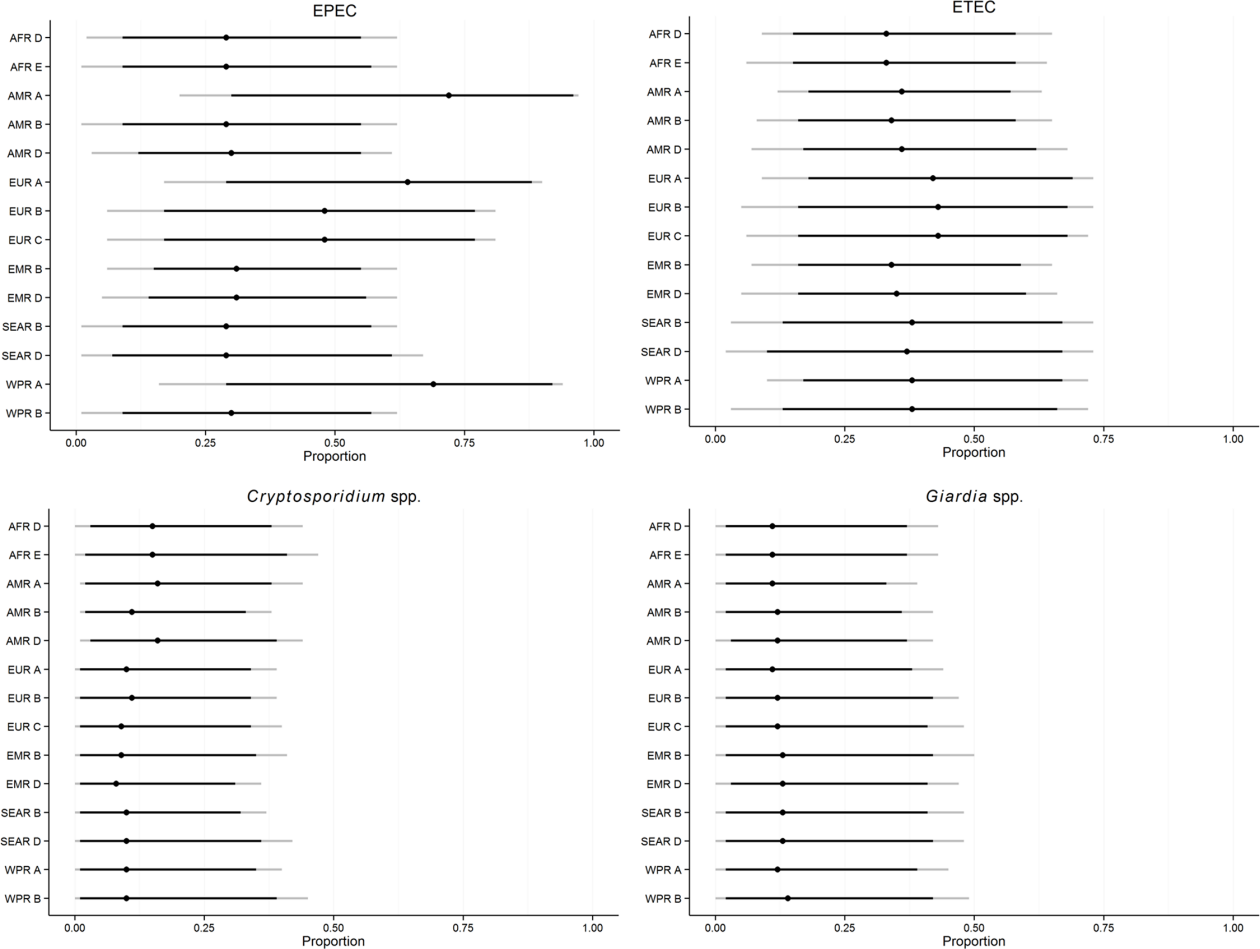
Subregional estimates of the proportion of foodborne illnesses caused by enteropathogenic *E*. (EPEC), enterotoxigenic *E*. *coli* (ETEC), *Cryptosporidium* spp. and *Giardia* spp. Indicated on the line plot are the 2.5^th^, 5^th^, 50^th^, 95^th^ and 97,5^th^ percentiles.

Fig 4 shows the subregional estimates of the proportion foodborne for enteropathogenic *E*. *coli* (EPEC), enterotoxigenic *E*. *coli* (ETEC), *Cryptosporidium* spp. and *Giardia* spp. The estimates for EPEC are seen to follow the same pattern as described above with the foodborne route being assessed to be more important in developed subregions. In developing subregions in the African, American and Eastern Mediterranean regions (AFR, AMR and EMR), water was identified as the most important transmission route. For ETEC, the estimated foodborne proportions were quite similar for all subregions with medians ranging from 0.33 to 0.43 (Table 6), but the foodborne route was only assessed by experts to be the more important route in European subregions. For *Cryptosporidium* spp. and *Giardia* spp., the foodborne proportions were also quite similar across subregions, but generally considered less important with medians below 0.20 (Table 6). Human-to-human and waterborne transmission were the more important routes for these infections in all subregions.

**Fig 5:**
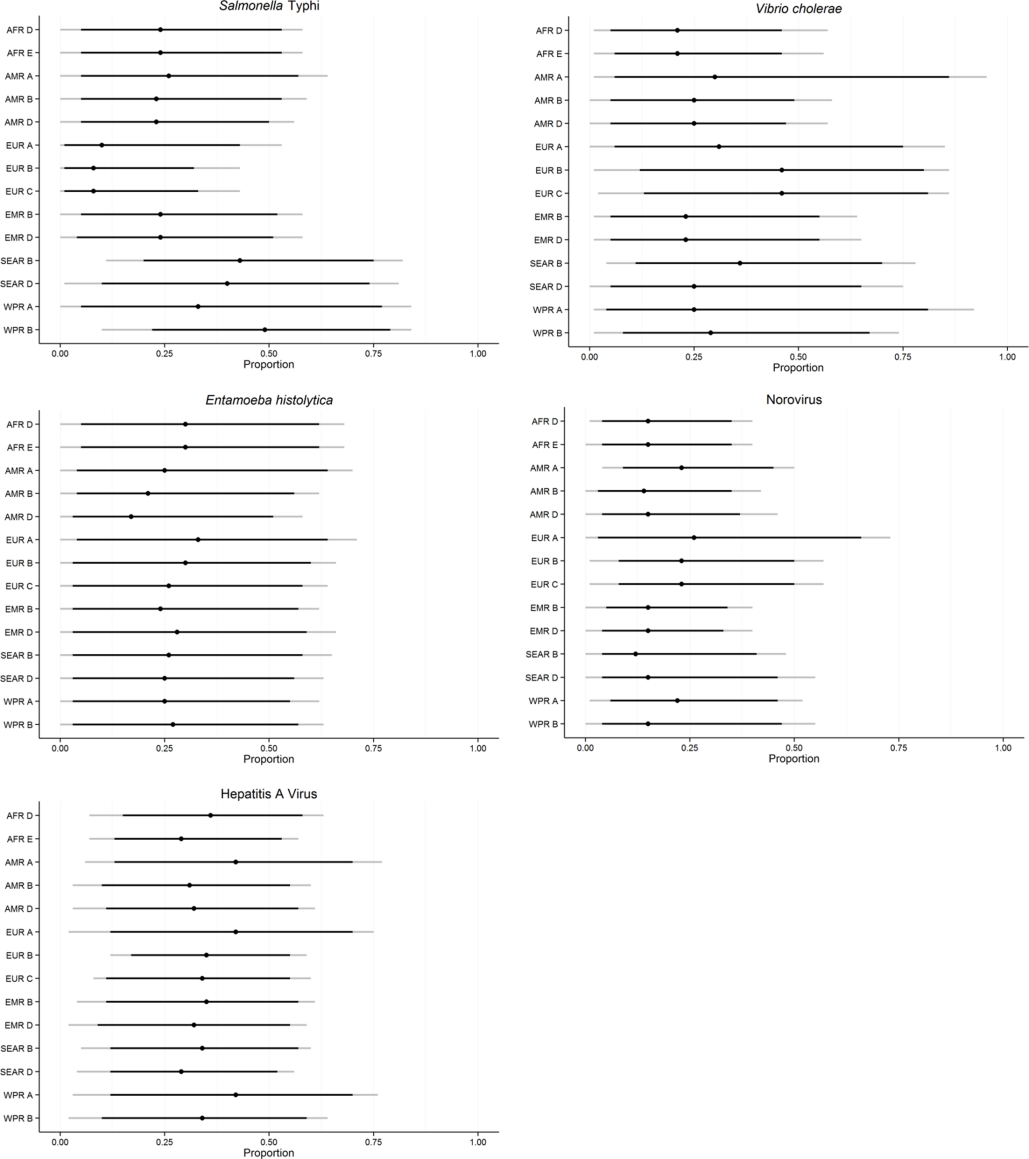
Subregional estimates of the proportion of foodborne illnesses caused by *Salmonella* Typhi, *Vibrio cholerae*, *Entamoeba histolytica*, Norovirus, and Hepatitis A virus. Indicated on the line plot are the 2.5^th^, 5^th^, 50^th^, 95^th^ and 97,5^th^ percentiles.

Fig 5 shows the subregional estimates of the proportion foodborne for *Salmonella* Typhi, *Vibrio cholerae*, *Entamoeba histolytica*, Norovirus, and Hepatitis A virus. Overall, foodborne infections were not assessed by the experts to be the more important routes in the majority of subregions. Exceptions were Hepatitis A infections, where foodborne and human-to-human transmission were evaluated equally important in most subregions, and *S*. Typhi, where foodborne and waterborne infections were assessed equally important in SEAR and WPR regions (Table 7). Human-to-human transmission was identified as the main exposure route for Norovirus and *E*. *histolytica* in most subregions, whereas waterborne transmission was estimated to be the main transmission route for *V*. *cholerae* infections (Table 7).

**Fig 6:**
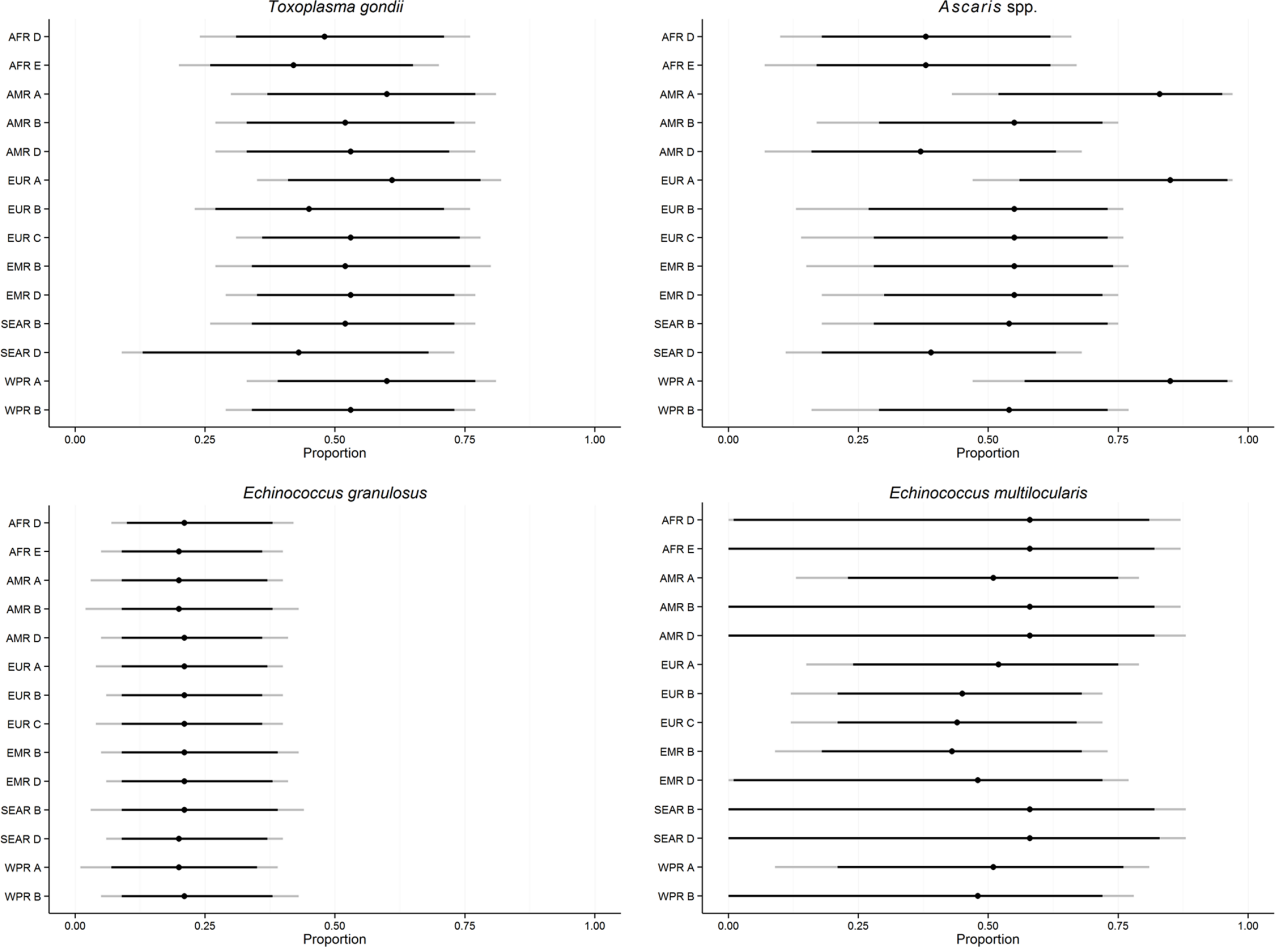
Subregional estimates of the proportion of foodborne illnesses caused by *Toxoplasma gondii*, *Ascaris* spp., *Echinococcus granulosus*, and *Echinococcus multilocularis*. Indicated on the line plot are the 2.5^th^, 5^th^, 50^th^, 95^th^ and 97,5^th^ percentiles.

Fig 6 shows the subregional estimates of the proportion foodborne for *Toxoplasma gondii*, *Echinococcus multilocularis*, *Echinococcus granulosus* and *Ascaris* spp. The foodborne route was assessed by the experts to be the most important transmission route for *T*. *gondii* and *Ascaris* spp. in most subregions, but there was a clear tendency for soil to increase in relative importance in less developed subregions (subregions D and E) (Table 8). Specifically for *Ascaris* spp., the foodborne route was assessed to be particularly important in developed subregions (subregions A). There was only little geographical variation between the median estimates for each of the transmission pathways for the two *Echinococcus* species. For *E*. *granulosus*, animal contact was clearly believed to be the most important route with medians just over 0.50. For *E*. *multilocularis*, the foodborne route was considered most important with medians ranging from 0.43 in EMR B to 0.58 in AFR D and E, AMR B and D, and SEAR B and D, but the estimates had very large uncertainty ranges (Table 8).

**Fig 7:**
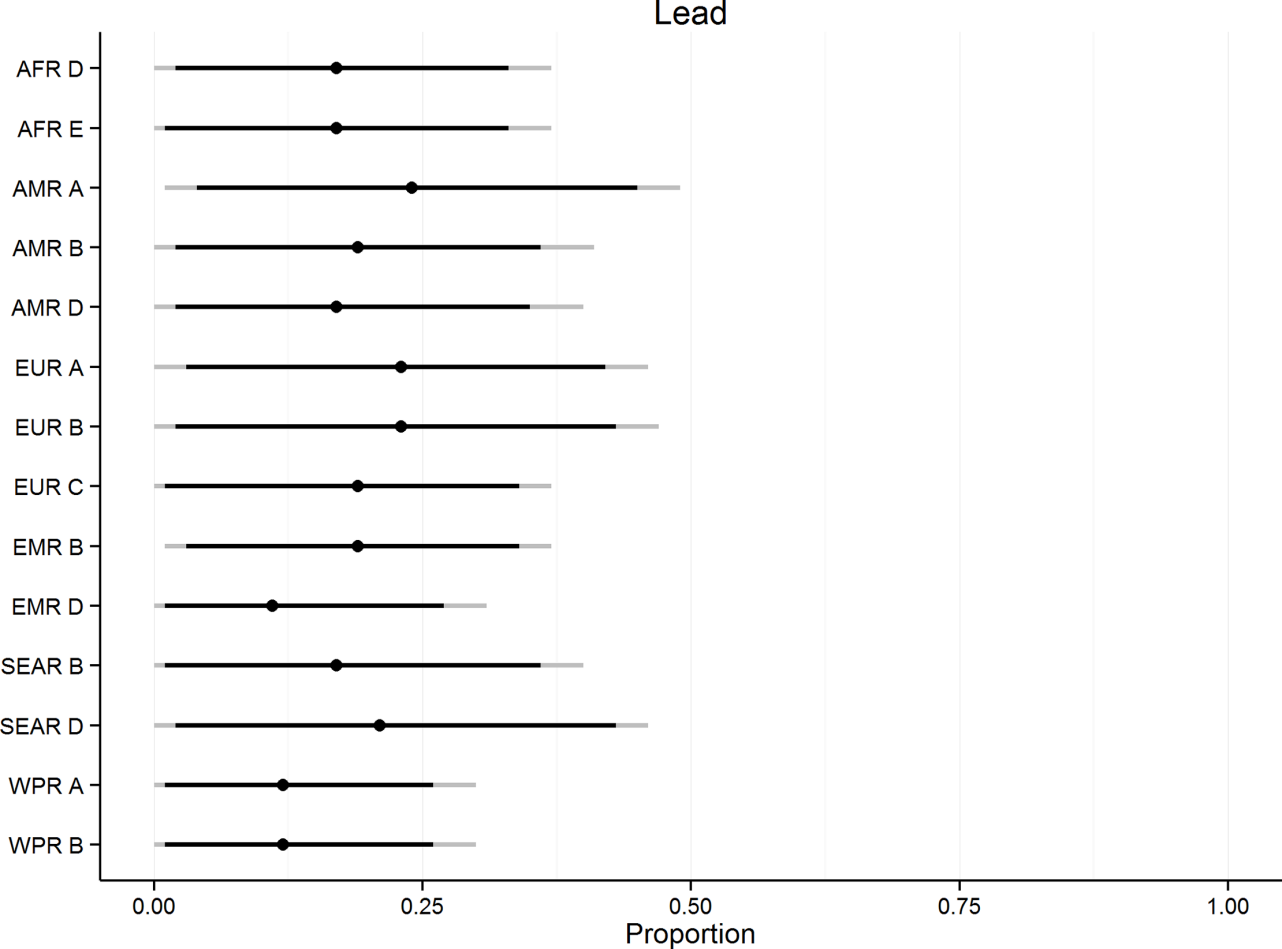
Subregional estimates of the proportion of disease caused by foodborne exposure to lead. Indicated on the line plot are the 2.5^th^, 5^th^, 50^th^, 95^th^ and 97,5^th^ percentiles.

**Fig 8:**
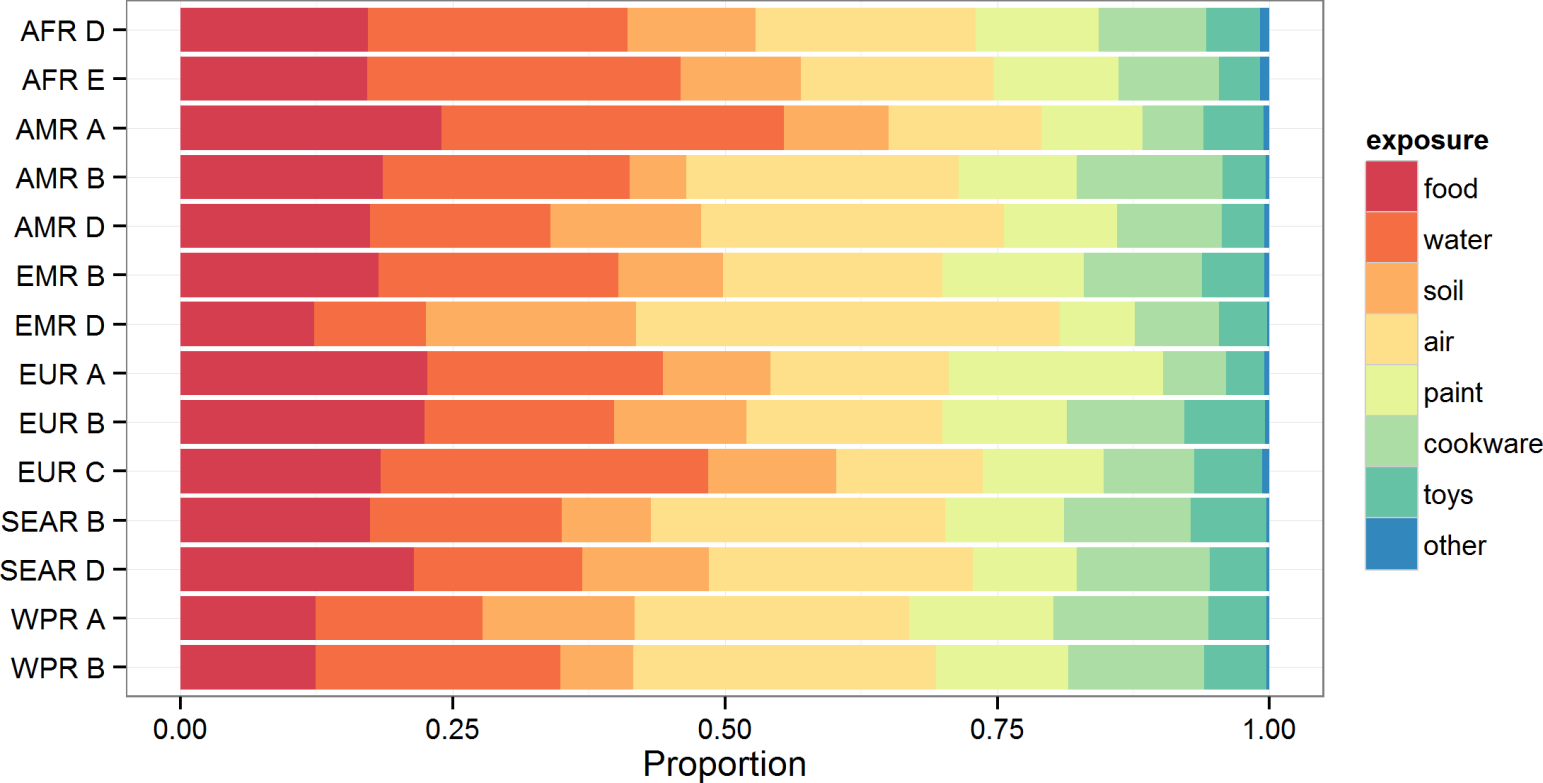
Subregional estimates (medians) of the proportion of disease caused by exposure to lead through eight different exposure routes.

Fig 7 shows the subregional estimates of the foodborne proportion for lead exposure. Water, food and air exposure were the main transmission routes indicated by the experts with some subregional differences (Table 9). The foodborne route was assessed to be the most important only in two subregions in Europe. Air was assessed to be the main exposure route in seven of the 14 subregions and water in four regions. Soil, paint, cookware/pottery/glassware and toys were in comparison only found to be of minor importance in the majority of subregions (Fig 8).

## Discussion

In this study, we present the results of the first world-wide study on the contribution of contaminated food and other exposure routes to human disease caused by 18 major microbiological hazards and a chemical hazard. The study highlights the importance of the foodborne route of transmission for these hazards and–when combined with estimates of incidence, severity, duration and mortality–allows estimation of the burden of foodborne disease [48]. Attempting to estimate foodborne transmission at the subregional level is an ambitious goal. However, this was vital given the geographically localized nature of exposure to many pathogens. These new findings are important due to the global nature of the estimation, the number of experts participating, and the rigorous approach taken to assessing and including expert performance in the final estimates.

We were unable to identify epidemiological studies in the literature that delineate and quantify the importance of each transmission pathway as investigated in this study. This makes it difficult to formally validate the outcomes of the expert elicitation. Still, a discussion of summary findings in the context of other scientific knowledge may be of value.

The hazards can be grouped in several categories with respect to their major pathways. For *Campylobacter* spp., non-typhoidal *Salmonella*, STEC, *T*. *gondii*, and *E*. *multilocularis*, the foodborne route was considered the most important route in all subregions. These pathogens are all zoonotic and known to have one or more animal reservoirs. The zoonotic nature of these organisms is also reflected in experts’ judgments by the identification of direct contact with animals as an important transmission route as well. Other pathogens with animal reservoir include *E*. *granulosus* and *Brucella* spp., and here direct contact with animals was considered equally or more important than food as routes of transmission.

As described in the results section for several pathogens, there was a clear pattern that the experts considered the foodborne route less important in low- and middle-income subregions, where other routes (animal contact, water and soil) were believed to contribute relatively more when compared to high-income subregions. This is consistent with data showing lower levels of access to improved water and sanitation in less developed regions as compared to high-income countries. This ranking of subregions across different pathogens provides some confidence in the results, as the estimates were done independently and partly by different experts.

An expert elicitation was used to estimate source attribution parameters not only because of the lack of globally consistent data on which to base such estimates, but also a general lack of data and research on source attribution in most parts of the world. The generally wide uncertainty bounds provided by the expert elicitation in this study are presumed to reflect both uncertainty and variation, where uncertainty arises due to the epistemic sparseness of hard evidence data, or the presence of conflicting evidence, on the contribution of different transmission pathways, and variation reflects the experts’ beliefs on aleatory variations between countries within any given subregion. A study operating with smaller regions or at country level may have reduced the uncertainty due to variation.

There exist a few recent national studies that estimate the proportion of illnesses attributable to the foodborne route for specific infectious diseases [40, 41, 42, 44, 59, 60]. Table 10 provides the main results from these studies. Four of the six studies used some kind of formal expert elicitation, where enrolled experts were asked to provide a central estimate and their uncertainty bounds around this. The estimates published by Gkoga et al. [59] and Scallan et al. [60] were derived by the authors by a synthesis of data from different public health surveillance systems and the literature. Studies also differed on the following two aspects that may explain some of the differences seen: 1) whether the study only addressed domestically acquired cases, and 2) whether more exposure routes than just the foodborne route were addressed. For illnesses with a high proportion of travel-related illness, and where the relative importance of exposure routes differs between domestically acquired and travel-related illness (e.g. illnesses caused by *S*. Typhi and *V*. *cholera*), the attribution estimates must be expected to differ between studies including travel-related illness and those only addressing domestically acquired illness. Only two of the national studies included more exposure routes than the foodborne [40, 41]. Asking experts to assign attribution estimates to all potential exposure routes and not just the foodborne has been discussed to result in relative lower estimates for the foodborne route [40], because experts are faced with providing estimates (and uncertainty bounds around these) for exposure routes that they may not otherwise have considered important, if they had only been asked about the foodborne route. Although, no overall trend of this can be seen from Table 10, it may be the case for hazards in regions that typically are thought of as being associated primarily with foodborne exposure (e.g. non-typhoidal *Salmonella* spp. and *Campylobacter* spp.). Finally, this study differed from the national studies by eliciting regional estimates, which could be interpreted as a weighted average across all countries in the subregion and thereby differ from country-specific estimates from the same subregion. As all six national studies were conducted in developed countries, we compare the results only with the results from the relevant subregions (i.e. EUR A, AMR A and WPR A) in this study.

**Table 10:** Percent of illness acquired through the foodborne transmission route for six national studies and this study ^a^.

	*Havelaar et al., [40]*	*Gkogka et al., [59]*	*This study*	*Ravel et al., [42]*	*Scallan et al., [60]*	*This study*	*Lake et al., [41]*	*Vally et al., [44]*	*This study*
Country/subregion*	NL	GR	EUR A	CA	USA	AMR A	NZ	AU	WPR A
**Period**	2006	1996–2006	2010	2008	2010	2010	2005	2010	2010
**Method**	Formal expert elicitation	Derived by the authors ^b^^)^	Formal expert elicitation	Formal expert elicitation	Derived by the authors ^b^^)^	Formal expert elicitation	Formal expert elicitation	Formal expert elicitation	Formal expert elicitation
**Only domestically acquired cases**	yes	depended on the data used	No	yes	yes	no	no	yes	no
**Hazards**									
* Brucella* spp.	-	84 (50–100)	66 (23–90)	-	50 (40–60)	75 (28–93)	-	-	-
* Campylobacter* spp.	42 (16–84)	55 (30–80)	76 (44–93)	68 (54–82)	80 (73–86)	73 (38–91)	56 (26–82)	76 (70–80)	68 (40–89)
* Cryptosporidium* spp.	12 (0–20)	5.6 (5.6–8)	10 (0–39)	9 (3–16)	8 (6–12)	16 (1–44)	-	-	-
* Entamoeba histolytica*	-	50 (10–100)	33 (0–71)	-	-	-	-	-	-
* *Enteropathogenic *E*. *coli*	-	-	-	-	-	-	-	24 (10–49)	69 (16–94)
* *Enterotoxigenic *E*. *coli*	-	-	-	-	100 (99–100)^c^^)^	36 (12–63)	-	24 (10–49)	38 (10–72)
* Giardia* spp.	13 (0–24)	10 (5–30)	11 (0–44)	-	7 (5–10)	11 (0–39)	-	-	-
* *Hepatitis A	11 (0–20)	8 (5–11)	42 (2–75)	-	6 (4–16)	42 (6–77)	-	12 (7–20)	42 (3–76)
* *Non-typhoidal *Salmonella* spp.	55 (32–88)	95 (55–95)	76 (47–94)	80 (68–92)	94 (91–96)	73 (38–91)	60 (18–83)	71 (65–75)	74 (45–93)
* *Norovirus	17 (16–47)	-	26 (0–73)	31 (14–48)	26 (19–35)	23 (4–50)	39 (8–64)	17 (5–30)	22 (1–52)
* Salmonella* Typhi	-	80 (55–95)	10 (0–53)	-	100 (76–100)	26 (0–64)	-	-	-
* *Shiga toxin-producing *E*. *coli*	42 (21–78)	51 (40–90)	60 (26–83)	76 (60–91)	82 (75–87)	59 (19–84)	40 (6–95)	55 (30–75)	57 (25–82)
* Shigella* spp.	-	10 (8.2–31)	7 (0–46)	18 (7–29)	31 (23–40)	12 (0–46)	-	11 (5–20)	13 (0–50)
* Toxoplasma gondii*	56 (26–88)	50 (30–63)	61 (35–82)	-	50 (40–60)	60 (30–81)	-	-	-
* Vibrio cholerae*	-	-	-	82 (66–98)	100 (99–100)	30 (1–95)	-	-	-

^a^ This table presents a measure of central tendency with its associated uncertainty bound from each study. Because studies differ in how they measure central tendency and uncertainty, we cannot label the columns with a single heading. Measures include: this study (median, 90% credibility interval (CI)); Havelaar et al. [40] (mean, 90% CI); Gkogka et al. [59](median, min-max); Ravel et al. [42] (mean, 95% CI); Scallan et al. [60] (mean, 90% CI); Lake et al. [41] (mean, 95% CI); Valley et al. [44] (median, 95% CI).

^b^ These estimates were derived by a synthesis of data from different public health surveillance systems and the literature.

^c^ Only ETEC cases reported as part of foodborne outbreaks were included in the study by Scallan et al. [60]. Consequently the proportion foodborne was per definition 100% and cannot be readily compared with the estimate in this study, which considers infections acquired from all transmission routes.

*Country/region abbreviations: NL = The Netherlands, GR = Greece, CA = Canada, USA = United States of America, NZ = New Zealand, AU = Australia. AMR A = Region of the Americas, Stratum A: very low child and adult mortality, EUR A = European Region, Stratum A: very low child and adult mortality, WPR = Western Pacific Region, Stratum A: very low child and adult mortality,

For the zoonotic pathogens, particularly non-typhoid *Salmonella* spp., the estimates are agreeing better and the uncertainty ranges tend to be relatively narrower than for pathogens with a human reservoir (e.g. Hepatitis A virus, *S*. Typhi and *V*. *cholerae*). As discussed above some of the differences observed may occur because we are comparing studies including only the foodborne route with studies including more exposure routes.

For Hepatitis A virus there is a strong divregence between the national studies and this study, where the proportion foodborne is estimated to be less, but at the same level in the four national studies that investigated this pathogen. This difference cannot be readily explained, but it should be noted that there was also disagreement between the experts in this study, where three of the six experts serving on the Hepatitis A virus panel provided estimates in line with those published for the national studies (see S3 File), whereas the remaining three experts provided considerably higher estimates. The differences could not be associated with the experts’ country of residence. The divergence between the experts is reflected in the uncertainty bounds for the estimates, which are quite wide and encompass the estimates from the national studies.

For *S*. Typhi and *V*. *cholerae*, the estimates from the national studies are higher than those found in this study. One explanation could be that the national studies [42, 59, 60] were only attributing domestically acquired cases. In the study by Scallan et al. [60], the proportion foodborne for *S*. Typhi was estimated based on data from 17 domestic outbreaks reported in a 19-year period, where 13 outbreaks were confirmed foodborne and 4 outbreaks were of unknown origin. The same study included also only data from domestically acquired cases of *V*. *cholerae*, and as around 70% of all *V*. *cholerae* and 67% of all *S*. Typhi cases were estimated to be travel related, including all cases could decrease the proportion of foodborne illness significantly. Infections with *S*. Typhi and *V*. *cholerae* are typically linked with contaminated water sources and poor hygiene in developing regions [24], thus these transmission routes are likely to be relatively more important among cases that have been travelling to these regions. This factor is probably reflected in the attribution results in this study.

The operational definition of different transmission routes, in particular the food and waterborne routes, will affect attribution estimates. Hazards transmitted by multiple routes can ‘change’ source or vehicle during the transmission from primary source to humans, meaning that the burden of illness caused by a particular hazard attributed to a specific transmission route may vary, depending on the point of attribution chosen [4, 7]. The choice of point of attribution seems particularly critical for delineating foodborne and waterborne diseases. This is because water is itself ingested just as a food, is used for irrigation of food plants, for washing and cleaning of food during preparation and constitutes an essential ingredient in many food products. In addition and particularly related to zoonotic diseases, the water source is often contaminated by an animal reservoir, including food-producing animals. Another situation relates to the consumption of water-based foods such as shellfish harvested from areas where the water contains pathogenic organisms, such as *Vibrio* spp. or enteric viruses. The burden related to the consumption of foods that have had contact with contaminated water at some stage of the production may, therefore, be attributed to either food or water depending on the point of attribution. It is clear that there exists no single ‘right’ way of delineating the foodborne route from other transmission routes; however, it is critical that point of attribution be clearly defined. That definition should depend on the objective and focus of the specific study, and could involve many factors, including the foodborne hazard in question, the food production systems and processing routines, the geographical occurrence of the hazard, sanitation and hygiene in the region, and consumption patterns. If the point of attribution is clearly defined, then additional modeling or further research can be used to adjust attribution to exposure at other points of interest in the transmission chain. If the point of attribution is not unambiguously defined, then not only are the results of the study unclear, but they will be difficult to use to model other relationships in the transmission chain.

**Fig 9:**
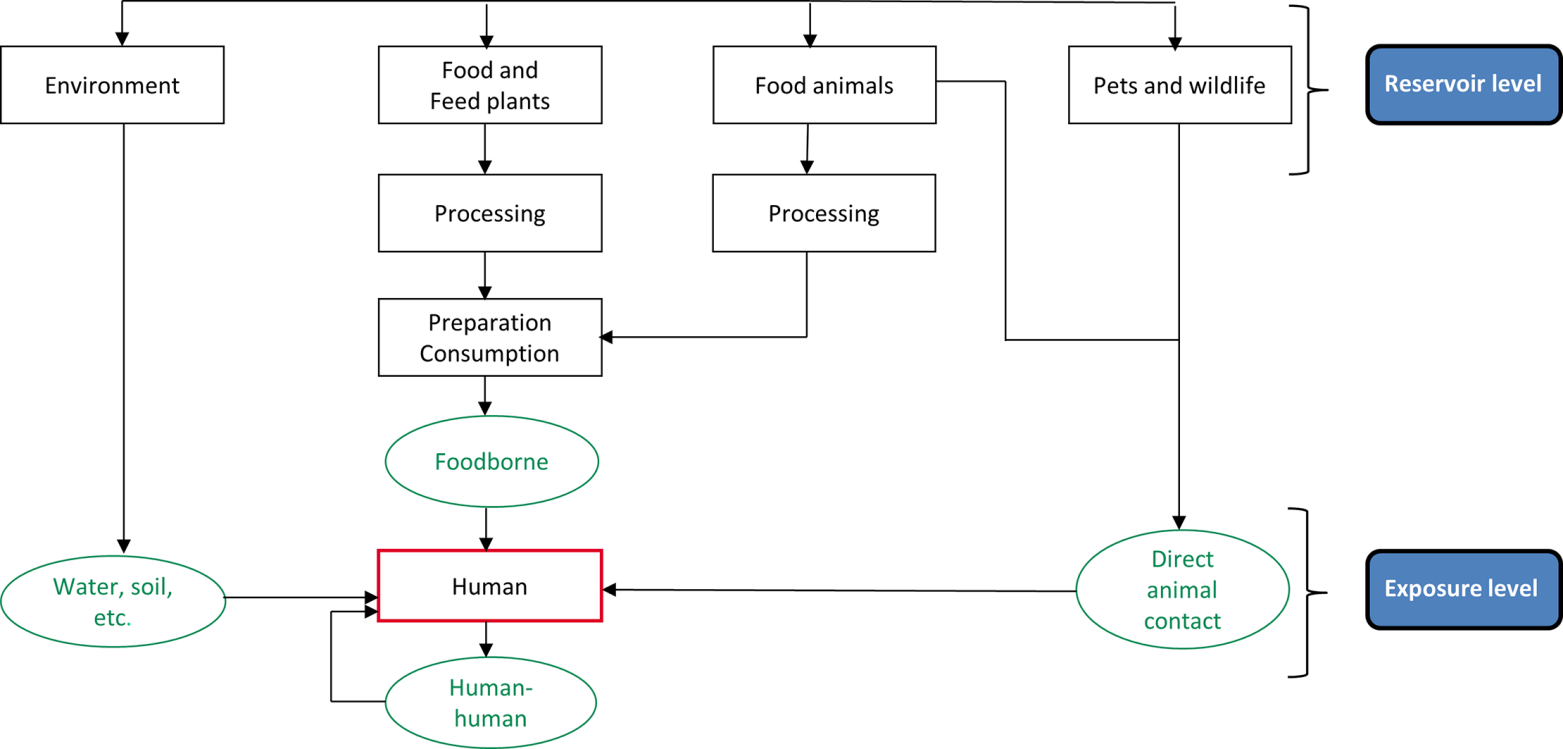
Major transmission routes of human foodborne diseases illustrating two points of attribution: the reservoir level and the exposure level.

FERG agreed that the point of human exposure was the most simple and understandable point of attribution to be used across all hazards for delineating the major transmission routes (Fig 9). FERG recognises that for other purposes, e.g. for risk management, other points of attribution may be more appropriate (e.g. primary production, processing and retail, or preparation). This said, the FERG definition of point of attribution for major transmission routes directly links attribution to disease incidence, as it is the end of the transmission chain. Further modeling can then be used to work backward from exposure to identify the important points of contamination.

In this study, we identified potential experts through peer-nomination, for the purpose of enumerating parameter uncertainties through structured expert judgment. It is important to recognize that the goal of a structured expert judgment is not to characterize the characteristics of the group of experts in some sense, but to obtain uncertainty quantifications of target variables which are statistically accurate and informative. The degree to which this goal is realized is assessed objectively by referencing elicited target uncertainties to the experts’ performances in judging similar, factual realizations of calibration variables in the subject matter field. This empirical validation of experts' (and combined experts') uncertainty quantifications is what distinguishes the Classical Model approach from surveys or statistical sampling [61]. When compared with the usual markers of professional qualifications, such as e.g. publication records [62], a formalized assessment of the uncertainty judgment capabilities of experts using calibration variables has also been demonstrated to be a better predictor of expert performance. Structured expert judgment studies are, therefore, not as sensitive to selection bias and low response rates as other types of surveys. In our study, we approached 299 potential experts and ended up with a final pool of 72 (24%), which is actually a fair response rate compared to population surveys, for example. The response rate of those specialists—confirmed as having appropriate expertise–who committed to participate in the study by forwarding their CV, DOI, etc., was 70%. The motives for some people declining to participate were not specifically asked for, but most of those giving a reason indicated lack of time. A few declined because they did not perceive themselves experts in the field. The reason given by the majority for not finally submitting responses to the target questionnaire, even though they had gone through the interview, was also time constraint. Although some selection bias cannot be ruled out absolutely, we do not believe that it has had major impact on the study results due to the formalized basis of the elicitation process and objective judgement pooling methodology.

Future research into source attribution should focus on developing more data-driven approaches, combining information from different sources in a single analytical framework. Ideally, this would include intervention studies examining the contribution of food to different diseases, along with source attribution studies based on highly specific genomic information from pathogens. To provide more consistent and comparable data, it is important that there is international harmonization of definitions, groupings, protocols, and data analysis methods.

## Conclusion

We provide for the first time global estimates for the proportions of diseases due to selected hazards that are attributable to food and to other major transmission routes. These estimates are essential for quantifying disease burdens within the FERG framework, and facilitates the estimation of the global burden of foodborne diseases [48] While some gaps exist, we believe the estimates presented here are the best current source of guidance to support decision makers when allocating resources for control and intervention, and for future research initiatives.

## Supporting Information

S1 File Supporting information on the Cooke’s Classical Model. 2015 Jan 14.
(PDF)

S2 File Seed question for microbiological and chemical hazards. (PDF)

S3 File Data file including anonymised responses from all experts. The Excel file includes a sheet for each hazard.
(XLSX)

## Abbreviations

FERG: Foodborne Disease Burden Epidemiology Reference Group
FBDs: Foodborne diseases
SATF FERG: Source Attribution Task Force
DOI: Declaration of Interests
SEJ: Structured Expert Judgment
