# Supplementary material for: World Health Organization Estimates of the Relative Contributions of Food to the Burden of Disease Due to Selected Foodborne Hazards: A Structured Expert Elicitation
Source: PLoS One. 2016 Jan 19;11(1):e0145839. doi: 10.1371/journal.pone.0145839 (PMC4718673; doi:10.1371/journal.pone.0145839)
Supplement: S2 File — (PDF) [file pone.0145839.s002.pdf]

Global Burden of Foodborne Disease
Expert Elicitation on the Sources of Foodborne Diseases

Sandra Hoffmann, Willy Aspinall, Roger Cooke, Tine Hald, Arie Havelaar
for the

World Health Organization
Foodborne Epidemiology Reference Group

August 2013

Seed questions for microbiological hazards

Table SI-1.1. Overview of seed questions for each hazard group. “x”: question included in expert performance analysis.” –“: question asked, but not included because data were not yet available at the time of analysis. Blank: question not included.

		Hazards							
**Question category**	**Question Number**	**Diarrheal Developed**	**Diarrheal Developing**	***Brucella* spp.**	**Hepatitis A virus**	**Intestinal protozoa**	***Toxoplasma gondii***	***Echino-coccus* spp.**	***Ascaris* spp.**
**Food supply**	**1.1**	x	x	x	x	x	x	x	x
	**1.2**	x	x	x	x	x	x	x	x
	**1.3**	-	-	-	-	-	-	-	-
	**1.4**	x	x	x	x	x	x	x	x
**Under-5 years mortality rate**	**2.1**	x	x	x		x		x	
	**2.2**		x		x	x	x		x
	**2.3**	x	x	x	x	x	x	x	x
**Access to improved water and Sanitation**	**3.1**	x	x	x	x	x	x	x	x
	**3.2**	x	x	x	x	x	x	x	x
	**3.3**	x	x	x	x	x	x	x	x
**Disease surveillance**	**4.1.1**			x					
	**4.1.2**		x		x	x		x	x
	**4.1.3**	x							
	**4.2.1**					x		x	x
	**4.2.2**	x	x				x		
	**4.2.3**				x				
	**4.2.4**			-					
	**4.3.1**					-	-	-	-
	**4.3.2**				-				
	**4.4.1**	-	-	-			-	-	-
	**4.4.2**				-				
	**4.5.1**	x	x	x					
	**4.5.2**					x	x	x	
	**4.6.1**	x							

**Systematic review questions**	**5.1**	x	x	x	x	x	x	x	x
	**5.2**	x	x	x	x	x	x	x	x
	**5.3**	x	x	x	x				
	**5.4**					x			
	**5.5**		x				x	x	x

1. Questions about food supply

1.1. Background: Food supply sources are changing in many countries. The Food and Agricultural Organization's Food Balance Sheet data allows calculation of the change in the percentage of a country's meat supply that was imported over time.

Among individual countries in the Eastern Mediterranean WHO Region (EMR B and EMR D), what was the largest *national* percentage point *decrease* from 2000 to 2009 in the proportion of meat supply (tonnes per year) that was imported rather than produced domestically? [for all panels]

Please express your answer in absolute terms.

Your response

	___________	___________	___________
	low	*best*	*high*
Percentile	(5th)	(50th)	(95th)

1.2. Background: Food supply sources are changing in many countries. The Food and Agricultural Organization's Food Balance Sheet data allows calculation of the change in the percentage of a WHO sub-region's vegetable supply (tonnes per year) that was imported over time.

Among all WHO sub-regions, what was the *largest regional* percentage point *increase* from 2000 to 2009 in the proportion of regional vegetable supply (tonnes) that was imported rather than produced domestically?

Please express your answer in absolute terms.

Your response

	___________	___________	___________
	low	*best*	*high*
Percentile	(5th)	(50th)	(95th)

1.3. The Food and Agricultural Organization's Food Balance Sheet data allows calculation of the percentage of a WHO sub-region's vegetable supply (tonnes per year) that was imported.

Among all WHO sub-regions, in 2010 what was the proportion of regional vegetable supply (tonnes) that was imported rather than produced domestically in the WHO sub-region with the highest such percentage?

Your response

	___________	___________	___________
	low	*best*	*high*
Percentile	(5th)	(50th)	(95th)

1.4. One available measure of the importance of meat in diets is meat as a percentage of the total food supply available for human consumption (kcal/capita/day).

Based on FAO Food Balance Sheet data, think of the countries in the Western Pacific Region (WPR A and WPR B) with the highest and lowest percentage of total national food supply (*kcal per capita per day*) available for human consumption that was meat in 2009. In 2009, what was the difference between the percentage of total national food supply available for human consumption that was meat in these two countries?

Please express your answer as a positive percentage point difference.

Your response

	___________	___________	___________
	low	*best*	*high*
Percentile	(5th)	(50th)	(95th)

2. Questions about under-5 years mortality

Background: The World Health Organization (WHO) began estimating cause-specific mortality of children under 5 years of age in the early 2000s. They now have estimates through 2010 by country and by WHO sub-region. The WHO provides estimates of the number of deaths from all causes per 1000 children under the age of 5. They also report estimates of the percentage of these deaths that are due to diarrhea.

2.1. Based on WHO's estimates, think of the country in the WHO Africa Region that had the largest percentage point decrease from 2000 to 2010 in all-cause under-5 mortality that was due to diarrhea. What was that percentage point *decrease?* You might want to look at the list of countries in the WHO Africa Region (AFR D and AFR E sub-regions).

Please express the change in absolute terms your answer.

Your response

	___________	___________	___________
	low	*best*	*high*
Percentile	(5th)	(50th)	(95th)

2.2. Based on WHO's estimates, think of the country in WHO's South East Asia (SEAR) region that had the highest national percentage of all-cause mortality in children under 5 that was due to diarrhea in 2010. What was that percentage? You might want to look at the list of countries in the WHO South East Asia Region (SEAR B and SEAR D sub-regions).

Your response

	___________	___________	___________
	low	*best*	*high*
Percentile	(5th)	(50th)	(95th)

2.3. WHO estimates of child mortality are given for individual countries. Based on WHO's estimates, for each WHO sub-region, think about the average percentage point change from 2000 to 2010 in the national percentage of all-cause mortality in children under 5 that was due to diarrhea. What was that average percentage change in the WHO sub-region with the largest such change?

Express your answer in absolute terms.

Your response

	___________	___________	___________
	low	*best*	*high*
Percentile	(5th)	(50th)	(95th)

3. Questions about change in access to improved water or improved sanitation

Background for questions 3.1. and 3.2: The World Health Organization and United Nations Childrens Fund, Joint Measurement Programme (JMP) has published estimates of percent of national population with access to improved sanitatidon for most countries in the world since 1990. Access to improved sanitation facilities is defined as "the percentage of national population with at least adequate access to excreta disposal facilities that can effectively prevent human, animal, and insect contact with excreta. Improved facilities range from simple but protected pit latrines to flush toilets with a sewerage connection. To be effective, facilities must be correctly constructed and properly maintained." (World Bank, http://data.worldbank.org/indicator/SH.H2O.SAFE.UR.ZS).
Data Source: jmp water and sanitation data_ 6 28 2013 wssinfo_data.xlsx

3.1. Access to improved sanitation increased at a regional level in all WHO regions from 1990 to 2010. Focus on the following WHO sub-regions: [AFR D, AFR E, AMR B, AMR D, EMR B, EMR D, EUR B, EUR C, SEAR B, SEAR D, and WPR B]. This group include all WHO sub-regions except "A" WHO sub-regions (those with very low child and adult mortality rates), for example, EUR A is excluded.

Based on the JMP data, for each of the above sub-regions, one could calculate the average national percentage point increase from 1990 to 2010 in each sub-region in access to improved sanitations. For example, one could calculate the average national increase in the percentage of population with access to sanitation among nations in the WHO Africa D (AFR D) region.

Think of the sub-region listed above that had the largest *average* national increase from 1990 to 2010 in the percentage of national population with access to sanitation. What was this increase?

Please express your answer in positive percentage points.

Your response

	___________	___________	___________
	low	*best*	*high*
Percentile	(5th)	(50th)	(95th)

3.2. Comparing all countries worldwide in 2010, think of the country that the UN Joint Measurement Programme reported as having the lowest percent of its population with access to improved sanitation. What percent of the population in that country had access to improved sanitation in 2010?

Your response

	___________	___________	___________
	low	*best*	*high*
Percentile	(5th)	(50th)	(95th)

3.3. Background:

The World Health Organization and United Nations Childrens Fund, Joint Measurement Programme (JMP) has published estimates of percent of national population with access to improved drinking water for most countries in the world since 1990. Access to improved drinking water is defined as the "percentage of the population with reasonable access to an adequate amount of water from an improved source, such as a household connection, public standpipe, borehole, protected well or spring, and rainwater collection. Reasonable access is defined as the availability of at least 20 liters a person a day from a source within one kilometer of the dwelling." (World Bank, Development Indicators, http://data.worldbank.org/indicator/SH.H2O.SAFE.UR.ZS).
Data source: jmp water and sanitation data_ 6 28 2013 wssinfo_data.xlsx

3.3. Comparing all countries worldwide, think of the country that the UN Joint Measurement Programme reported as having the lowest percent of its national population with access to improved drinking water in 1990. What was the percentage point increase from 1990 to 2000 in the percent of national population with access to improved drinking water in that country?

Your response

	___________	___________	___________
	low	*best*	*high*
Percentile	(5th)	(50th)	(95th)

4. Questions Based on Disease Surveillance Data

4.1.1 Background: All EU member states collect active surveillance data on several major zoonoses and on foodborne outbreaks (Zoonoses Directive 2003/99/EC). They report this information to the European Food Safety Authority (EFSA) annually. EFSA , in collaboration with the European Centre for Disease Prevention and Control, publish an annual summary of this data, usually released early in the calendar year. The latest EFSA summary report published 2011 data (EFSA Feb. 2013). The report anticipated for release in early 2014 will report 2012 surveillance data. The following questions refer to the EFSA reports.

What will be the number of confirmed human cases of brucellosis in 2012 in all EU member states as will be reported in EFSA's annual report?

Your response

	___________	___________	___________
	low	*best*	*high*
Percentile	(5th)	(50th)	(95th)

Did you know the answer to this question because you are working on the forthcoming European estimates? ___________

4.1.2. Background: All EU member states collect active surveillance data on several major zoonoses and on foodborne outbreaks (Zoonoses Directive 2003/99/EC). They report this information to the European Food Safety Authority (EFSA) annually. EFSA , in collaboration with the European Centre for Disease Prevention and Control, publish an annual summary of this data, usually released early in the calendar year. The latest EFSA summary report published 2011 data (EFSA Feb. 2013). The report anticipated for release in early 2014 will report 2012 surveillance data. The following questions refer to the EFSA reports.

4.1.2. What will be the rate per 100,000 population of laboratory confirmed human cases of campylobacteriosis in 2012 in all EU member states as reported in EFSA's annual report?

Your response

	___________	___________	___________
	low	*best*	*high*
Percentile	(5th)	(50th)	(95th)

Did you know the answer to this question because you are working on the forthcoming European estimates? ___________

4.1.3. Background: "Beginning in 2008, EU member states implemented *Salmonella* control programmes for *S. Enteritidis* and *S. Typhimurium* in laying flocks of *Gallus gallus* providing eggs intended for human consumption in accordance with Regulation (EC) No. 2160/2003." (EU Summary report: Trends and Sources of Zoonoses, Zoonotic Agents and Food-borne Outbreaks in 2010 (Feb. 2011)). Under this programme, laying flocks must be sampled for these pathogens every 15th week during the production period and results of these tests must be reported to EFSA annually. These results are included in the EFSA/ECDC annual report, "Trends and Sources of Zoonoses, Zoonotic Agents and Food-borne Outbreaks". The following questions refer to these annual EFSA/ECDC reports.

What will be the percent positive among these samples of laying flocks for all reporting EU member states in 2012?

Your response

	___________	___________	___________
	low	*best*	*high*
Percentile	(5th)	(50th)	(95th)

Did you know the answer to this question because you are working on the forthcoming European estimates? ___________

4.2.1 Background: Since 1996, the U.S. Foodborne Diseases Active Surveillance Network, or FoodNet, has been tracking trends for infections commonly transmitted through food. This active surveillance is currently done in 10 of the 50 U.S. states. CDC uses FoodNet surveillance data to monitor trends in incidence of specific foodborne illnesses over time. Results are reported annually, usually in the spring. The most recent available data is for 2012. New estimates are released in early summer of each year.

Source: http://www.cdc.gov/foodnet/data/trends/tables/2012/table2a-b.html#table-2a

Based on active surveillance data from FoodNet, what will be the incidence of laboratory-confirmed human *Cryptosporidium* infections (infections per 100,000 population) in 2013?

Your response

	___________	___________	___________
	low	*best*	*high*
Percentile	(5th)	(50th)	(95th)

Did you know the answer to this question because you are working on these forthcoming estimates?
____

4.2.2. Background: Since 1996, the U.S. Foodborne Diseases Active Surveillance Network, or FoodNet, has been tracking trends for infections commonly transmitted through food. This active surveillance is currently done in 10 of the 50 U.S. states. CDC uses FoodNet surveillance data to monitor trends in incidence of specific foodborne illnesses over time. Results are reported annually, usually in the spring. The most recent available data is for 2012. New estimates are released in early summer of each year.

4.2.2. Based on active surveillance data from FoodNet, what will be the rate per 100,000 population of laboratory-confirmed human cases of STEC (including STEC O157 and STEC non-O157) in 2013?

Your response

	___________	___________	___________
	low	*best*	*high*
Percentile	(5th)	(50th)	(95th)

Did you know the answer to this question because you are working on these forthcoming estimates? ___________

4.2.3. Background: The U.S. "National Notifiable Disease Surveillance System" (NNDSS), tracks contagious diseases that laboratory professionals and doctors are required to report to the state or territorial public health agency. These agencies voluntarily submit the information to NNDSS, which the Centers for Disease Control and Prevention (CDC) oversees. The general system has been in existence since 1878. Notifiable disease surveillance is "passive" (i.e., the investigator at CDC waits for disease reports from those required to report) and is susceptible to underreporting. Foodborne diseases that are reportable include botulism, cholera, hepatitis A, hemolytic uremic syndrome (HUS), listeriosis (*Listeria*), salmonellosis (*Salmonella*), Shiga Toxin-producing *Escherichia coli* (STEC) infections, shigellosis (*Shigella*), and vibriosis." Annual reports are issued in July. The most recently available data is for 2011 published in MMRW July 5, 2013.

The annual number of human cases of acute Hepatitis A reported to U.S. CDC through the NNDSS passive surveillance system has declined markedly over the past decade. What was the percent decline from 2000 to 2010 in the annual number of cases of acute hepatitis A reported to U.S. CDC through the NNDSS system?

Your response

	___________	___________	___________
	low	*best*	*high*
Percentile	(5th)	(50th)	(95th)

4.2.4. Background: The U.S. Animal Health Protection Service (APHIS) in cooperation with state departments of health collect surveillance data on cases of *M. bovis* and *Brucella* infection. In October of each year APHIS publishes annual totals of illnesses for the U.S. federal fiscal year (October through September). What will be the number of laboratory confirmed *M. bovis* cases in the U.S. between October 1, 2013 and September 31, 2013?

Your response

	___________	___________	___________
	low	*best*	*high*
Percentile	(5th)	(50th)	(95th)

Did you know the answer to this question because you are working on these forthcoming estimates?
____

4.3.1 The New Zealand Ministry of Health publishes annual reports on surveillance of notifiable diseases in New Zealand. New reports are released in April. The most recent data is for 2012 cases. Source: ESR, New Zealand Public Health Observatory, Notifiable Diseases, http://www.nzpho.org.nz/NotifiableDisease.aspx.

What will be the rate of human giardiasis cases per 100,000 population in New Zealand in 2013?

Your response

	___________	___________	___________
	low	*best*	*high*
Percentile	(5th)	(50th)	(95th)

Did you know the answer to this question because you are working on these forthcoming estimates?
____

4.3.2 Background: The New Zealand Ministry of Health publishes annual reports on surveillance of notifiable diseases in New Zealand. New reports are released in April. The most recent data is for 2012 cases. Source: ESR, New Zealand Public Health Observatory, Notifiable Diseases, http://www.nzpho.org.nz/NotifiableDisease.aspx.

What will be the rate of human cases of hepatitis A infections per 100,000 population in 2013 in New Zealand?

Your response

	___________	___________	___________
	low	*best*	*high*
Percentile	(5th)	(50th)	(95th)

Did you know the answer to this question because you are working on these forthcoming estimates?
____

4.4.1. Background: All Australian states and territories require doctors and/or pathology laboratories to report cases of infectious diseases that are important to public health to public health authorities. Western Australia is the only jurisdiction where laboratory notification is not mandatory under legislation, although most laboratories still notify the health department by agreement. OzFoodNet aggregates this data and reports cases and case rates per 100,000 population for *Campylobacter,* non-typhoidal *Salmonella, Listeria*, shiga toxin producing *Escherichia coli* infections, typhoid, and *Shigella* infections. OzFoodNet also collects and reports information on gastrointestinal outbreaks. OzFoodNet publishes an annual report on these data usually in September. The most recent year that has been published is 2010, published in September 2012. Source: **www.ozfoodnet.gov.au.**

4.4.1. What will OzFoodNet report as the percent of human gastrointestinal outbreaks in Australia in 2011 that were associated with foodborne transmission?

Your response

	___________	___________	___________
	low	*best*	*high*
Percentile	(5th)	(50th)	(95th)

Did you know the answer to this question because you are working on these forthcoming estimates?
____

4.4.2. Background: All Australian states and territories require doctors and/or pathology laboratories to report cases of infectious diseases that are important to public health to public health authorities. Western Australia is the only jurisdiction where laboratory notification is not mandatory under legislation, although most laboratories still notify the health department by agreement. OzFoodNet aggregates this data and reports cases and case rates per 100,000 population for *Campylobacter,* non-typhoidal *Salmonella, Listeria*, shiga toxin producing *Escherichia coli* infections, typhoid, and *Shigella* infections. OzFoodNet also collects and reports information on gastrointestinal outbreaks. OzFoodNet publishes an annual report on these data usually in September. The most recent year that has been published is 2010, published in September 2012. Source: **www.ozfoodnet.gov.au.**

What will OzFoodNet report as the rate of human hepatitis A cases per 100,000 population in Australia in 2011?

Your response

	___________	___________	___________
	low	*best*	*high*
Percentile	(5th)	(50th)	(95th)

Did you know the answer to this question because you are working on these forthcoming estimates? ___________

4.5.1. Background: Health Canada reports annual case rates for notifiable diseases reported through the Canadian Notifiable Disease Surveillance System (CNDSS). Data has been released through 2011. 2012 case rates will be released in early 2014.

4.5.1. What was the change in the national Canadian case rate (cases per 100,000 population) of campylobacteriosis) reported from the CNDSS from 2000 to 2010?

Your response

	___________	___________	___________
	low	*best*	*high*
Percentile	(5th)	(50th)	(95th)

4.5.2 Background: Health Canada reports annual case rates for notifiable diseases reported through the Canadian Notifiable Disease Surveillance System (CNDSS). Data has been released through 2011. 2012 case rates will be released in early 2014.

What was the change in the national Canadian case rate (cases per 100,000 population) of giardiasis reported from the CNDSS from 2000 to 2010?

Your response

	___________	___________	___________
	low	*best*	*high*
Percentile	(5th)	(50th)	(95th)

4.6.1. Background: The Japanese Ministry of Health, Labour and Welfare" publishes data on the annual national number of foodborne outbreaks and foodborne outbreak associated cases caused by bacteria, viruses and parasites and specific bacteria, viruses and parasites. This allows the Ministry to calculate the percentage of foodborne outbreaks and outbreak associated cases that are associated with particular pathogens each year.

4.6.1. What was the percentage point change from the percent of total foodborne outbreaks associated with *Salmonella* in Japan in 2000 to the percent associated with *Salmonella* in 2010?

Your response

	___________	___________	___________
	low	*best*	*high*
Percentile	(5th)	(50th)	(95th)

5. Questions based on Systematic reviews

5.1. Soares Magalhãesa et al. (2011) collected data on drinking water sources, sanitation, household demographics and household location from recent demographic health surveys in Burkina Faso (2003), Ghana (2003) and Mali (2006) and parasitology survey data collected by the Schistosomiasis Control Initiative from school-aged children in Burkina Faso (2007), Ghana (2008), and Mali (2007). They used this to estimate the population attributable fraction of hookworm due to living in a home with a soil floor in West Africa, i.e., the percent of hookworm disease that is caused by living in a home with a dirt floor. What was their estimate, as a percentage?

Your Response

	___________	___________	___________
	low	*best*	*high*
Percentile	(5th)	(50th)	(95th)

Do you know the answer to this question with substantial certainty because you are worked on this study? ___________

5.2. Background: Fewtrell et al (2005) conducted a systematic review and meta-analysis to compare the evidence of relative effectiveness of improvements in drinking water, sanitation facilities, and hygiene practices in less developed countries in reducing diarrheal illness. The study included only published studies that reported diarrheal illness not associated with an outbreak as the health outcome. Meta-analysis was used to estimate the relative risk of diarrheal illness with and without each of these interventions and in combination.

Hygiene interventions were defined as "those that included hygiene and health education and the encouragement of specific behaviours, such as handwashing." (p. 43). "Sanitation interventions were those that provided some means of excreta disposal, usually latrines (either public or household). Water supply inteventions included the provision of a new or improved water supply, or improved distribution (such as the installation of a hand pump or household connection). This could be at the public level or household level. Water quality interventions were related to the provision of water treatment for the removal of microbial contaminants, either at the source or at the household level. Multiple inteventions were those which introduced water, sanitation and hygiene or health education) elements to the study population.

Fewtrell et al (2005) include a meta-analysis of 5 studies was used to estimate the relative risk of diarrheal illness with and without multiple interventions. What was the estimated relative risk?

Remember relative risk is between 0 and 1.

Your response

	___________	___________	___________
	low	*best*	*high*
Percentile	(5th)	(50th)	(95th)

5.3. Background: Wang et al. 2007 analyses 2 review articles and official Chinese reports of bacterial foodborne disease outbreaks to identify foodborne illness outbreaks in China between 1995 and 2005. The analysis identified 1082 outbreaks caused by foodborne bacteria. (Wang et al. 2007. Analysis of bacterial foodborne disease outbreaks in China between 1994 and 2005, FEMS Immunol Med Microbiol 51: 8–13.)

What percent of all identified outbreak -related cases were due to *Salmonella* in Wang et al. 2007?

Your response

	___________	___________	___________
	low	*best*	*high*
Percentile	(5th)	(50th)	(95th)

5.4. Background: Hörman et al. (2004) conducted a meta-analysis of original studies published prior to 2004 on the epidemiology of *Giardia* and *Cryptospordium* in Nordic countries. Meta-analysis using a random effects model with a DerSimonian-Laird estimator was used to estimate prevalence and annual incidence of these parasites in Denmark, Finland, Norway and Sweden. (Hörman et al., 2004. Meta-analysis in assessment of the prevalence and annual incidence of Giardia spp. and Cryptosporidium spp. infections in humans in the Nordic countries. International Journal for Parasitology 34: 1337–1346.)

What was the rate of reporting *Giardia* symptomatic cases in Finland (registered cases/unregistered symptomatic cases) per 100,000 according to estimates by Hörman et al. (2004)?

Your response

	___________	___________	___________
	low	*best*	*high*
Percentile	(5th)	(50th)	(95th)

5.5. Background: Baldursson and Karanis (2011) published a comprehensive review of worldwide of outbreaks caused by waterborne parasitic protozoa occurring and published between January 2004 and December 2010. In their search, the authors defined parasitic protozoa as including *Cryptospordium, Giardia lamblia, Cyclospora, Blastocystis, Entamoeba, Acanthamoeba, Toxoplasma,* microsporidia, *Sarcocystis, Naegleria, Balantidium coli,* and *Isospora.* (Baldursson and Karanis. 2011. Waterborne transmission of protozoan parasites: Review of worldwide outbreaks. Water Research. 45: 6603-14.)

What percent of waterborne parasitic protozoan outbreaks reported worldwide in published form between January 2004 and December 2010 do Baldursson and Karanis (2011) find to be due to *Cryptosporidium spp?*

Your response

	___________	___________	___________
	low	*best*	*high*
Percentile	(5th)	(50th)	(95th)

Global Burden of Foodborne Disease
Expert Elicitation on the Sources of Foodborne Diseases

Sandra Hoffmann, Willy Aspinall, Roger Cooke, Tine Hald, Arie Havelaar
for the

World Health Organization
Foodborne Epidemiology Reference Group

November 2013

Seed questions for chemical hazards

Lead

1. The U.S. Centers for Disease Control and Prevention (CDC) annually surveys about 5,000 individuals in the U.S. as part of the National Health and Nutrition Examination Survey (NHANES). The survey includes both interviews and physical examinations.

What was the geometric mean blood lead concentration for all participants ages 1 year and older in the 2007-2008 U.S. NHANES survey?

Please express your answer as positive micrograms per deciliter (µg/dL).[1]

Your response

	___________	___________	___________
	low	*best*	*high*
Percentile	(5th)	(50th)	(95th)

2. In 2010, the UN Environmental Programme published a final review of scientific information on lead. In this review, they reported data from Japan's 2005 submission of data on the contribution of different foods to dietary exposure to lead in the Japanese population.

What percent of dietary lead exposure in the Japanese population came from rice consumption?

Your response

	___________	___________	___________
	low	*best*	*high*
Percentile	(5th)	(50th)	(95th)

3. In 2010, the UN Environmental Programme published a final review of scientific information on lead. In this review they reported mean blood lead levels for children in a number of countries.

What did the UNEP Final Review of Scientific Information on Lead report in 2010 as the mean blood lead level for children in Nigeria?

Please express your answer as positive micrograms per deciliter (µg/dL).

Your response

	___________	___________	___________
	low	*best*	*high*
Percentile	(5th)	(50th)	(95th)

[1] Please note, we have experienced instability in Word©'s representation of the symbol for the Greek letter "mu". As a result, we have written out measurements in full each time they are used. If there is disagreement between the abbreviation and the written measure, assume the measurement written in full is correct.

Arsenic

4. In 2009, the European Food Safety Authority published a Scientific Opinion on Arsenic in Food. The opinion included estimates of the concentrations of total arsenic in food based on a call for data from EU member states and Norway from 2003 to 2008. What was the mean concentration of total arsenic in bran and germ reported in the 2009 EFSA Scientific Opinion on Arsenic in Food? Please express your answer in milligrams per kilogram (mg/kg).

Your response

	___________	___________	___________
	low	*best*	*high*
Percentile	(5th)	(50th)	(95th)

5. In 2011, the Joint FAO/WHO Expert Committee on Food Additives (JECFA) published a review of scientific information on dietary exposure to arsenic around the world. JECFA referenced a study by Amini et al. (2008) that "modeled the probability that arsenic concentrations would be above 10 micrograms/liter (µg/L) in shallow groundwater" for many countries around the world. Based on this study, JECFA (2011) included a table that reports the percent of land area in different countries that is predicted to have a greater than 75% likelihood of having groundwater with arsenic concentrations of more than 10 micrograms/liter (µg/L). How many times higher was this percent area in Cambodia than that in Argentina?

Your response

	___________	___________	___________
	low	*best*	*high*
Percentile	(5th)	(50th)	(95th)

6. In 2009, the European Food Safety Authority published a Scientific Opinion on Arsenic in Food. The report estimates daily dietary exposure of Europeans to inorganic arsenic in various foods. What was the estimated daily dietary exposure to inorganic arsenic of high consumers of algae-based products in Europe? Express your answers in micrograms per kilogram body weight per day (µg /kg b.w. per day).

Your response

	___________	___________	___________
	low	*best*	*high*
Percentile	(5th)	(50th)	(95th)

Cadmium

7. In 2011, the Joint FAO/WHO Expert Committee on Food Additives (JECFA) published a safety evaluation of cadmium and other chemicals. What did JECFA report as was the difference between the upper and lower bound dietary exposure to cadmium in Australia (2000-2001) for adult males age 25-34 years of age? Please express your answer in micrograms per kilogram body weight per month (µg /kg b.w. per month).

Your response

	___________	___________	___________
	low	*best*	*high*
Percentile	(5th)	(50th)	(95th)

8. In 2011, the Joint FAO/WHO Expert Committee on Food Additives (JECFA) published a safety evaluation of cadmium and other chemicals. JECFA estimated mean regional dietary cadmium exposure for 12 regions in China and for the country as a whole. What was the difference between the mean regional dietary exposure to cadmium in Sichuan, the region with the highest exposure, and the national mean exposure estimate? Express your answer in micrograms per kilogram body weight per month (µg /kg b.w. per month).

Your response

	___________	___________	___________
	low	*best*	*high*
Percentile	(5th)	(50th)	(95th)

9. The UNEP, Final Review of Scientific Information on Cadmium (Dec. 2010) discussed exposure to cadmium from multiple exposure routes. According to this report, in Finland how many times higher was the daily dietary intake of cadmium than the average daily exposure to cadmium via water?

Your response

	___________	___________	___________
	low	*best*	*high*
Percentile	(5th)	(50th)	(95th)

Dietary Patterns and Food Supply

10. One available measure of the importance of rice in diets is rice as a percentage of the total national food supply available for human consumption. National food supply available for human consumption is measured in kilograms per capita per year.

Based on this FAO Food Balance Sheet data, in 2009 what was the mean percentage of rice in the national food supply available for human consumption for countries in the WHO South East Asia Region, sub-region B (SEAR B)?

Your response

	___________	___________	___________
	low	*best*	*high*
Percentile	(5th)	(50th)	(95th)

11. One available measure of the importance of fish in diets is fish as a percentage of the total national food supply available for human consumption. National food supply available for human consumption is measured in kilograms per capita per year.

Based on this FAO Food Balance Sheet data, in 2009 what was the mean percentage of fish and seafood in the national food supply available for human consumption for countries in the WHO Western Pacific Region, sub-region B (WPR B)?

Your response

	___________	___________	___________
	low	*best*	*high*
Percentile	(5th)	(50th)	(95th)

12. The Food and Agricultural Organization's Food Balance Sheet data allows calculation of the percentage of the national fish supply that was imported. National fish supply is measured in tonnes per year.

In 2009 what was the mean percentage of national fish supply that was imported rather than produced domestically for countries in the WHO Europe, sub-region A (EUR A)?

Your response

	___________	___________	___________
	low	*best*	*high*
Percentile	(5th)	(50th)	(95th)
